# Identifying Patients for Intensive Blood Pressure Treatment Based on Cognitive Benefit: A Secondary Analysis of the SPRINT Randomized Clinical Trial

**DOI:** 10.1001/jamanetworkopen.2023.14443

**Published:** 2023-05-19

**Authors:** Lama Ghazi, Jincheng Shen, Jian Ying, Catherine G. Derington, Jordana B. Cohen, Zachary A. Marcum, Jennifer S. Herrick, Jordan B. King, Alfred K. Cheung, Jeff D. Williamson, Nicholas M. Pajewski, Nick Bryan, Mark Supiano, Josh Sonnen, William S. Weintraub, Tom H. Greene, Adam P. Bress

**Affiliations:** 1 Department of Epidemiology, School of Public Health, University of Alabama at Birmingham; 2 Department of Family and Preventive Medicine, University of Utah, Salt Lake City; 3 Department of Internal Medicine, Spencer Fox Eccles School of Medicine, University of Utah, Salt Lake City; 4 Intermountain Healthcare Department of Population Health Sciences, Spencer Fox Eccles School of Medicine, University of Utah, Salt Lake City; 5 Department of Medicine, Renal-Electrolyte and Hypertension Division, Perelman School of Medicine at the University of Pennsylvania, Philadelphia; 6 Department of Biostatistics, Epidemiology, and Informatics, Perelman School of Medicine, University of Pennsylvania, Philadelphia; 7 Department of Pharmacy, University of Washington School of Pharmacy, Seattle; 8 George E. Wahlen Department of Veterans Affairs Medical Center, Salt Lake City, Utah; 9 Institute for Health Research, Kaiser Permanente Colorado, Aurora; 10 The Sticht Center for Healthy Aging and Alzheimer’s Prevention, Wake Forest School of Medicine, Winston-Salem, North Carolina; 11 Department of Biostatistics and Data Science, Wake Forest School of Medicine, Winston-Salem, North Carolina; 12 Department of Radiology, Perelman School of Medicine, University of Pennsylvania, Philadelphia; 13 Division of Geriatrics, University of Utah School of Medicine, and The Center on Aging, University of Utah, Salt Lake City; 14 Department of Pathology and Neurology and Neurosurgery, McGill University School of Medicine, Montreal, Quebec, Canada; 15 MedStar Health Research Institute and Georgetown University, Washington, DC

## Abstract

**Importance:**

Intensive vs standard treatment to lower systolic blood pressure (SBP) reduces risk of mild cognitive impairment (MCI) or dementia; however, the magnitude of cognitive benefit likely varies among patients.

**Objective:**

To estimate the magnitude of cognitive benefit of intensive vs standard systolic BP (SBP) treatment.

**Design, Setting, and Participants:**

In this ad hoc secondary analysis of the Systolic Blood Pressure Intervention Trial (SPRINT), 9361 randomized clinical trial participants 50 years or older with high cardiovascular risk but without a history of diabetes, stroke, or dementia were followed up. The SPRINT trial was conducted between November 1, 2010, and August 31, 2016, and the present analysis was completed on October 31, 2022.

**Intervention:**

Systolic blood pressure treatment to an intensive (<120 mm Hg) vs standard (<140 mm Hg) target.

**Main Outcomes and Measures:**

The primary outcome was a composite of adjudicated probable dementia or amnestic MCI.

**Results:**

A total of 7918 SPRINT participants were included in the analysis; 3989 were in the intensive treatment group (mean [SD] age, 67.9 [9.2] years; 2570 [64.4%] men; 1212 [30.4%] non-Hispanic Black) and 3929 were in the standard treatment group (mean [SD] age, 67.9 [9.4] years; 2570 [65.4%] men; 1249 [31.8%] non-Hispanic Black). Over a median follow-up of 4.13 (IQR, 3.50-5.88) years, there were 765 and 828 primary outcome events in the intensive treatment group and standard treatment group, respectively. Older age (hazard ratio [HR] per 1 SD, 1.87 [95% CI, 1.78-1.96]), Medicare enrollment (HR per 1 SD, 1.42 [95% CI, 1.35-1.49]), and higher baseline serum creatinine level (HR per 1 SD, 1.24 [95% CI, 1.19-1.29]) were associated with higher risk of the primary outcome, while better baseline cognitive functioning (HR per 1 SD, 0.43 [95% CI, 0.41-0.44]) and active employment status (HR per 1 SD, 0.44 [95% CI, 0.42-0.46]) were associated with lower risk of the primary outcome. Risk of the primary outcome by treatment goal was estimated accurately based on similar projected and observed absolute risk differences (C statistic = 0.79). Higher baseline risk for the primary outcome was associated with greater benefit (ie, larger absolute reduction of probable dementia or amnestic MCI) of intensive vs standard treatment across the full range of estimated baseline risk.

**Conclusions and Relevance:**

In this secondary analysis of the SPRINT trial, participants with higher baseline projected risk of probable dementia or amnestic MCI gained greater absolute cognitive benefit from intensive vs standard SBP treatment in a monotonic fashion.

**Trial Registration:**

ClinicalTrials.gov Identifier: NCT01206062

## Key Points

**Question:**

How does the magnitude of projected cognitive benefit from intensive vs standard systolic blood pressure (SBP) treatment vary across patients?

**Findings:**

In this secondary analysis of 7918 participants in the Systolic Blood Pressure Intervention Trial with a median follow-up of 4 years, participants with higher baseline risk of probable dementia or amnestic mild cognitive impairment (MCI) gained greater absolute cognitive benefit from intensive vs standard SBP treatment.

**Meaning:**

These findings suggest that intensive SBP treatment reduces the risk of combined MCI or dementia overall and to a greater degree among those with higher baseline risk of MCI or dementia in a monotonic fashion.

## Teaser

This secondary analysis of the randomized clinical Systolic Blood Pressure Intervention Trial (SPRINT) develops a model to estimate the patient-specific benefits of intensive vs standard systolic blood pressure (SBP) treatment among adults with high cardiovascular risk.

## Introduction

In the Systolic Blood Pressure Intervention Trial (SPRINT), intensive (systolic blood pressure [SBP] goal <120 mm Hg) vs standard (SBP goal <140 mm Hg) treatment reduced the risk of a composite outcome of mild cognitive impairment (MCI) and probable dementia, in addition to cardiovascular disease (CVD) and all-cause mortality.^1,2^ While Williamson et al^1^ have previously shown that intensive treatment reduced the risk of MCI or probable dementia overall on average, the study did not address treatment effect heterogeneity and which populations derive the greatest absolute benefit for prioritization. As we have previously seen, the magnitude of absolute benefit from intensive treatment for CVD outcomes was greatest for those at highest baseline CVD risk, and this population should initially be targeted.^3^ As such, cognitive benefit from intensive vs standard SBP treatment may vary among patients.

Detecting patient characteristics associated with the greatest magnitude of cognitive benefit from intensive vs standard SBP treatment may help prioritize patients, as the intensive treatment component requires additional health care resources (clinical time, office and laboratory visits, and medications) and increases the risk of mostly mild, transient treatment-related adverse events.^4^ Two broad classes of statistical approaches are currently being used to identify patient subgroups with higher or lower predicted magnitude of benefit from a treatment in a randomized clinical trial: effect modeling and risk modeling.^5^ Effect modeling uses regression with interaction terms between the randomized treatment and baseline covariates to identify subgroups with varying treatment effects, usually on a risk ratio or hazard ratio (HR) scale. In contrast, risk modeling explores how the absolute benefit of treatment (ie, the absolute risk reduction of cognitive outcomes), the most clinically relevant effect measure, varies as a function of the baseline risk of the outcome (a function of many baseline factors assessed simultaneously), which usually varies substantially within a clinical trial population. This approach has been termed *risk magnification* of the treatment effect. Unlike CVD risk, standardized, validated, and widely compatible risk prediction models for cognitive impairment are lacking. We developed an internal multivariable model to estimate patient-specific magnitude of benefits of intensive vs standard SBP treatment on cognitive outcomes in SPRINT participants using a risk-modeling approach.

## Methods

### SPRINT Design

The SPRINT design, rationale, and main results have been published previously,^1,2,6^ and the trial protocol is provided in Supplement 1. This study was reviewed and approved by the University of Utah Institutional Review Board. The study protocols for SPRINT and the included cohorts were approved by the institutional review boards at each participating institution, and all participants provided written informed consent. We followed the Consolidated Standards of Reporting Trials (CONSORT) reporting guideline for randomized clinical trials.

Briefly, SPRINT recruited participants 50 years or older with increased cardiovascular risk and an SBP between 130 to 180 mm Hg, depending on the number of antihypertensive medications used. Those with a history of stroke, heart failure, standing BP less than 110 mm Hg, diabetes, estimated glomerular filtration rate less than 20 mL/min/1.73m^2^, and dementia were excluded. Participants were randomized to target an intensive (SBP <120 mm Hg) or a standard (SBP <140 mm Hg) treatment goal. SPRINT enrolled patients between November 1, 2010, and March 31, 2013, and was completed on August 31, 2016. Cognitive assessments were continued through July 30, 2018, and the present analysis was completed on October 31, 2022.

### Study Population for the Current Analysis

**Figure 1.**
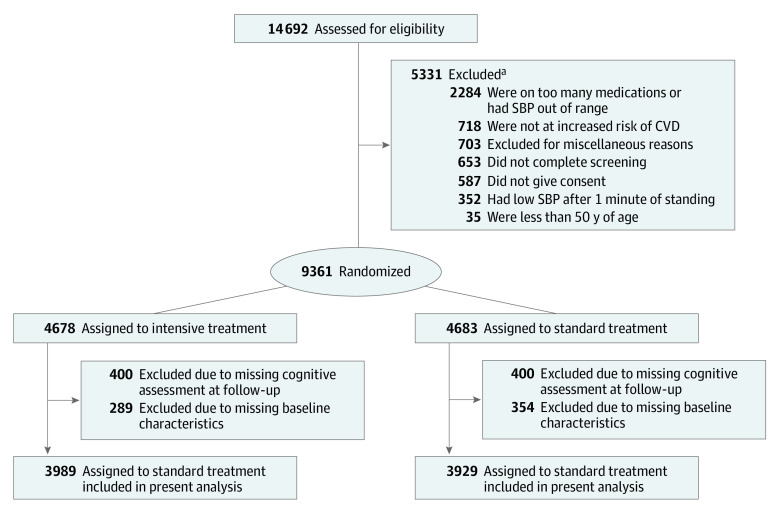
Participant Eligibility, Randomization, and Follow-up CVD indicates cardiovascular disease; SBP, systolic blood pressure. ^a^One participant contributed twice to the exclusion criteria.

We excluded participants without a cognitive assessment during follow-up (643 of 9631 randomized [6.7%]) or who had missing values of baseline covariates. Our study population included 7918 SPRINT participants, 3989 in the intensive treatment group and 3929 in the standard treatment group (Figure 1).

### Ascertainment of MCI and Probable Dementia

In SPRINT, cognitive screening assessments were administered at baseline, 2 and 4 years of follow up, study closeout (if it was >1 year from the 4-year visit), and an extended follow-up visit between October 1, 2017, and July 30, 2018 (eFigure 1 in Supplement 2). Assessments were performed by certified examiners at local study sites.

Cognitive assessment in SPRINT included a screening battery administered to all participants: (1) Montreal Cognitive Assessment for global cognitive function; (2) Logical Memory forms I and II subtests of the Wechsler Memory Scale for learning and memory; and (3) Digit Symbol Coding Test of the Wechsler Adult Intelligence Scale for processing speed.^7,8,9^ For participants scoring below prespecified cutoffs adjusted for age, race and ethnicity, culture, and educational level, an extended cognitive assessment battery was also administered. A Functional Activities Questionnaire of 10 items to measure functional abilities was administered to a preidentified proxy for participants with a low score on any of the screening tests^10^ (eMethods and eTable 1 in Supplement 2). For participants who could not be assessed in person during follow-up, a validated telephone battery test was administered. If participants scored at or below 31 on the Modified Telephone Interview for Cognitive Status, the participant’s proxy was administered the Functional Activities Questionnaire.^11^ However, if a participant died or was unable to communicate by telephone, a prespecified proxy was administered the Dementia Questionnaire.^12^ All participants were given standardized questionnaires of depressive status, perceived health status, quality of life, and self-reported medications, medical problems, and health habits (ie, smoking, alcohol use, physical activity).^13^ Cognitive status was adjudicated by 2 blinded experts using standardized classified criteria. Disagreements were discussed and adjudicated by a full panel. Participants were classified as having no cognitive impairment, MCI (amnestic or nonamnestic), probable dementia, or cannot classify.

### Outcomes

Our primary outcome for this analysis was a composite of adjudicated probable dementia or amnestic MCI. We chose to include amnestic MCI instead of any MCI for the following reasons. The definition of MCI in the original SPRINT protocol (ie, protocol-defined MCI) required 2 consecutive occurrences of MCI (amnestic or nonamnestic). For this analysis, to avoid conditioning the MCI outcome on future cognitive assessments and to include as many MCI events as possible, we chose to analyze the first occurrence of amnestic MCI, which has been consistently associated with the highest increased risk of progression to dementia.^14,15,16^ Secondary outcomes included occurrence of (1) probable dementia, amnestic MCI, or death; (2) probable dementia or death; and (3) probable dementia, protocol-defined MCI, or death. We also considered a broader definition of MCI as the first-time occurrence of any adjudicated MCI and assessed the occurrence of the composite outcome of protocol-defined MCI, probable dementia, or death.

### Covariates

To develop the multivariable model, we identified 58 candidate baseline covariates considered likely to be associated with cognitive function based on expert opinion (L.G., J.D.W., M.S., and A.P.B.) and published literature.^17,18,19,20,21,22,23,24,25,26^ These covariates comprised demographic, social, clinical, laboratory, and medication variables at the SPRINT baseline visit (ie, prerandomization). Race and ethnicity were self-reported in SPRINT, and these variables were included because previous trials have identified racial differences in cognitive outcomes, and this merited inclusion in the current studies. Additionally, the National Institutes of Health requires the inclusion of race and ethnicity in clinical trials. These groups included Hispanic, non-Hispanic Black, non-Hispanic White, and other (comprising American Indian and Alaska Native, Asian, Native Hawaiian or other Pacific Islander, and other race or ethnicity). For statistical purposes, race was categorized as Black vs non-Black participants, with the non-Black category comprising Hispanic, White, and other race categories. Additionally, clinicians with expertise in hypertension and dementia subject matter selected covariates that are likely to be available at the point of care—that is, at the time clinicians discuss decisions about intensive SBP treatment with their eligible patients (basic reduced set in eTable 2 in Supplement 2). We identified 3 different covariate sets to assess the information we can recover from our estimation model under a range of scenarios in clinical and research practice (eTable 2 in Supplement 2). The first covariate set (full set) included all candidate covariates. The second covariate set (augmented reduced set) included only covariates known to be associated with cognitive function regardless of whether these covariates are readily available in discrete data fields at the point of care. The final covariate set (basic reduced set) only included covariates that are available at the point of care and can be easily obtained in discrete data fields in the primary care setting. This prioritization was also informed by the national patient-centered clinical research network PCORnet Common Data Model variable list, version 6.0 (ie, representing structured data elements commonly available in US health system electronic health record databases).^27^

### Statistical Analysis

We described the association of each covariate with each of the cognitive outcomes using bar plots of HRs (expressed relative to 1-SD increases in the covariates). We used univariable Cox proportional hazards regressions of each outcome onto each covariate, separately.

#### Estimation Model Development

We fit a modified elastic-net Cox proportional hazards regression model for the projection of each outcome using the 3 covariate sets separately (full set, augmented reduced set, and basic reduced set).^28^ The elastic net is a machine learning method for regression analysis that seeks to provide accurate estimation from a large number of possibly correlated covariates (estimation variables) by balancing estimation accuracy with a term that penalizes excessively large regression coefficients based on correlation between the estimation variables. To allow for potential heterogeneity in the treatment effect of intensive vs standard treatment across the full range of baseline risk, we modified the standard elastic-net procedure to simultaneously estimate a numerical baseline risk score for each cognitive outcome as well as its interaction with the treatment groups (intensive vs standard). In this way, for each cognitive outcome and each of the 3 covariate sets, the analysis produced both a baseline risk score that estimates the overall risk of the cognitive outcome across the 2 treatment groups as well as the dose-response association between the baseline risk score and the treatment effect expressed as the difference in the absolute risk of the cognitive outcome between the treatment groups. To improve accuracy, our model requires that the difference in absolute risks must approach 0 as the baseline risk score approaches 0. We presented graphical displays of the association between the estimated risk difference and the baseline risk score under the modified elastic-net model (which we refer to as risk magnification plots) to illustrate the consequences of risk amplification on the treatment effect. All absolute risks used in computing the risk differences were estimated at 4.13 years, the median follow-up time in SPRINT for cognitive outcomes.

#### Adequacy of Estimation Models for the Risks of the Cognitive Outcomes

We evaluated the adequacy of our models for estimating risk of cognitive outcomes by providing standard measures of discrimination and calibration. The concordance (or C) statistic was used to assess discrimination, which evaluates the models’ ability to distinguish between patients with higher vs lower risks for the cognitive outcomes.^29,30^ A C statistic of 0.5 indicates random concordance, while a C statistic of approximately 0.8 is commonly thought of as a threshold of clinical relevant estimation. We visually displayed model calibration by comparing the observed risk of a given outcome (estimating directly using a Kaplan-Meier curve) with the mean estimated risk under the model across subgroups defined by the deciles of estimated risks. With these plots, we provided the mean of the absolute errors (MAE)^30,31^ (the absolute difference between the observed and estimated risks) across the 10 deciles as a numerical calibration measure. The smaller the MAE, the lower the error and the better the model calibration. We also provided *P* values from the Greenwood-Nam-D’Agostino test (GND),^30,31^ which assesses whether the deviation between the observed and estimated risks of the outcome under the model is larger than expected by chance. A nonsignificant GND *P* value (*P* > .05) indicated that the data were consistent with proper calibration.

#### Adequacy of the Estimation Models for Differences in Risk Between Treatment Groups

For each cognitive outcome and for each covariate set, we summarized the adequacy of the modified elastic-net model to estimate the risk differences in the cognitive outcome between treatment groups by dividing the cohort into tertiles of the estimated risk differences and comparing the observed risk differences (estimated from the Kaplan-Meier curves within tertiles at 4.13 years) to the estimated risk differences under the model. We report the observed risk differences in association with the estimated risk differences on the aforementioned risk amplification plots, where the 95% CIs for the observed risk differences are marked for all tertiles.

#### Evaluating Effect Modeling in Addition to Risk Modeling

We also tested a hybrid risk-modeling and effect-modeling approach by adding interaction terms between treatment assignment and prespecified subgroups to the elastic-net Cox proportional hazards regression models (eMethods in Supplement 2). These included age (<75 vs ≥75 years), sex (women vs men), race (Black vs non-Black), baseline MCI (with vs without), and baseline CVD (with vs without). This analysis addressed whether adding interactions of treatment group with these prespecified baseline factors improved the modified elastic-net Cox proportional hazards regression models’ ability to estimate cognitive treatment benefit. These analyses were repeated for all outcomes. Analyses were performed in SAS, version 9.4 (SAS Institute Inc), and R, version 3.5.1 (R Project for Statistical Computing). The R code to estimate cognitive benefit using baseline covariates is available elsewhere.^32^

## Results

Of 8451 SPRINT participants randomized with complete baseline and follow-up cognitive assessment data, 7918 were included in the present analysis. Of these, 3989 participants were included in the intensive treatment group (mean [SD] age, 67.9 [9.2] years; 2570 [64.4%] men and 1419 [35.6%] women; 1212 [30.4%] non-Hispanic Black and 2777 [69.6%] non-Black participants) and 3929 were included in the standard treatment group (mean [SD] age, 67.9 [9.4] years; 2570 [65.4%] men and 1359 [34.6%] women; 1249 [31.8%] non-Hispanic Black and 2680 [68.2%] non-Black participants) (Table 1). Participants in both the intensive and standard treatment groups had similar educational attainment, insurance coverage, medical history, and clinical and laboratory characteristics. Over a median follow up of 4.13 (IQR, 3.50-5.88) years, 765 participants in the intensive treatment group and 828 in the standard treatment group had probable dementia and amnestic MCI (Table 2). In the highest tertile of estimated benefit using the full data set, a projected value of −0.027 translates to 27 events prevented per 1000 persons treated with intensive vs standard treatment over 4.13 years of follow-up. A total of 854 participants in the intensive treatment group and 954 in the standard treatment group had probable dementia, amnestic MCI, or death (eTable 3 in Supplement 1). Moreover, 256 participants in the intensive treatment group and 314 in the standard treatment group had probable dementia or died (eTable 4 in Supplement 1). Additionally, 482 participants in the intensive treatment group and 574 in the standard treatment group had probable dementia or protocol-defined MCI or died (eTable 5 in Supplement 1). The difference between observed and mean estimated risks within tertiles of estimated cognitive benefit were minimal for all outcomes. For example, the difference between the elastic-net Cox proportional hazards regression estimated risk and observed risk for the first estimated benefit tertile using the full set was −0.014. This minimal difference indicates that our estimation model was able to project our outcome very close to the observed risk. Additionally, the difference between our estimated and observed model was similar whether we used the full set, augmented reduced set, or basic reduced set. As such, we could use a reduced set with a smaller number of covariates and expect similar performance from our estimation model. This remained true across all outcomes and covariate sets (Table 2 and eTables 3-5 in Supplement 2).

**Table 1.** Baseline Characteristics of SPRINT Participants Included for Model Derivation

Characteristic	Treatment group^a^	
	Intensive treatment (n = 3989)	Standard treatment (n = 3929)
Age, mean (SD), y	67.9 (9.2)	67.9 (9.4)
Sex		
Men	2570 (64.4)	2570 (65.4)
Women	1419 (35.6)	1359 (34.6)
Race and ethnicity		
Non-Hispanic Black	1212 (30.4)	1249 (31.8)
Non-Black^b^	2777 (69.6)	2680 (68.2)
Social and behavioral		
Lives with others	2871 (72.0)	2806 (71.4)
Has private insurance	1778 (44.6)	1656 (42.1)
Current smoker	519 (13.0)	502 (12.8)
Former smoker	1719 (43.1)	1681 (42.8)
Never smoker	1751 (43.9)	1746 (44.4)
Employed	854 (21.4)	850 (21.6)
Engages in vigorous physical activity (≥15 min/d)	2922 (73.3)	2912 (74.1)
Alcohol consumption, median No. of drinks/d (IQR)	0 (0-0.4)	0 (0-0.4)
Educational attainment		
Less than high school	338 (8.5)	354 (9.0)
High school graduate only	658 (16.5)	630 (16.0)
Post–high school graduate	1418 (35.5)	1394 (35.5)
College graduate or greater	1575 (39.5)	1551 (39.5)
Health insurance status^c^		
Uninsured	410 (10.3)	400 (10.2)
Medicare	2176 (54.6)	2185 (55.6)
Medicaid	241 (6.0)	259 (6.6)
Private	1778 (44.6)	1656 (42.1)
VA	815 (20.4)	785 (20.0)
Usual source of care		
Physicians’ office and/or outpatient clinic	3377 (84.7)	3348 (85.2)
Community health care facility or other	449 (11.3)	430 (10.9)
No usual source of care	163 (4.1)	151 (3.8)
Medical history		
Clinical CVD	624 (15.6)	602 (15.3)
Left ventricular hypertrophy	726 (18.2)	722 (18.4)
Dizziness when standing	161 (4.0)	161 (4.1)
History of coronary revascularization	381 (9.6)	361 (9.2)
History of depression (self-report)	714 (17.9)	709 (18.0)
Baseline cognitive assessments		
Montreal Cognitive Assessment, median (IQR) score^d^	24 (21-26)	23 (21-26)
Logical Memory form II, median (IQR) score^e^	9 (6-11)	8 (6-11)
Digit Symbol Coding Test, median (IQR) score^f^	51 (42-61)	51 (41-61)
Serial 7s, median (IQR) score^g^	3 (2-3)	3 (2-3)
Clinical and laboratory measurements, median (IQR)		
Systolic BP, mm Hg	138 (129-149)	139 (130-149)
Diastolic BP, mm Hg	78 (70-86)	78 (70-86)
Resting heart rate, beats/min	65 (58-73)	65 (58-74)
Serum potassium level, mg/dL	4.2 (3.9-4.5)	4.2 (3.9-4.4)
Serum creatinine level, mg/dL	1.0 (0.9-1.2)	1.0 (0.9-1.2)
Albumin to creatinine ratio, mg/g	9.5 (5.7-20.5)	9.4 (5.6-21.3)
Total cholesterol level, mg/dL	187 (161-215)	186 (161-214)
HDL cholesterol level, mg/dL	50 (43-60)	50 (43-60)
Triglyceride level, mg/dL	108 (77-150)	107 (77-153)
BMI	29.1 (26.0-33.1)	29.1 (26.0-32.8)
Serum glucose level, mg/dL	97 (91-105)	97 (91-105)
Medication use		
Aspirin	2144 (53.7)	2056 (52.3)
Statin	1716 (43.0)	1769 (45.0)
NSAID	1557 (39.0)	1429 (36.4)
Medication use		
Benzodiazepines	239 (6.0)	221 (5.6)
Anticholinergics	386 (9.7)	365 (9.3)
Antidepressants	476 (11.9)	475 (12.1)
No. of nonantihypertensive medications, median (IQR)	3 (1-5)	3 (1-5)
No. of antihypertensive medications, median (IQR)	2 (1-3)	2 (1-3)
ARB	851 (21.3)	852 (21.7)
ACE-I	1543 (38.7)	1440 (36.7)
DHP CCB	1187 (29.8)	1197 (30.5)
Non-DHP CCB	207 (5.2)	225 (5.7)
Thiazide diuretic	1585 (39.7)	1628 (41.4)
Loop diuretic	225 (5.6)	213 (5.4)
β-Blocker	1528 (38.3)	1372 (34.9)
α-Blocker	403 (10.1)	396 (10.1)
Other antihypertensive medication	387 (9.7)	414 (10.5)

Abbreviations: ACE-I, angiotensin-converting enzyme inhibitor; ARB, angiotensin II receptor blocker; BMI, body mass index (calculated as weight in kilograms divided by height in meters squared); BP, blood pressure; CCB, calcium channel blocker; CVD, cardiovascular disease; DHP, dihydropyridine; HDL, high-density lipoprotein; NSAID, nonsteroidal anti-inflammatory drug; VA, Department of Veterans Affairs.

SI conversion factors: To convert potassium to mmol/L, multiply by 1.0; to convert creatinine to μmol/L, multiply by 88.4; to convert total cholesterol to mmol/L, multiply by 0.0259; to convert HDL cholesterol to mmol/L, multiply by 0.0259; to convert triglycerides to mmol/L, multiply by 0.0113; to convert serum glucose to mmol/L, multiply by 0.0555.

^a^ Unless otherwise indicated, data are expressed as No. (%) of participants.

^b^ Includes Hispanic, non-Hispanic White, and other (American Indian or Alaska Native, Asian, Native Hawaiian or other Pacific Islander, and other race or ethnicity).

^c^ Participants were able to choose to more than 1 category.

^d^ Scores range from 0 to 30, with higher scores denoting better cognitive function. Subtest of the Wechsler Memory Scale.

^e^ Scores range from 0 to 14, with higher scores denoting better cognitive function. Subtest of the Wechsler Adult Intelligence Scale.

^f^ Scores range from 0 to 135, with higher scores denoting better cognitive function.

^g^ Scores range from 1 to 5, with higher scores indicating better cognitive function.

**Table 2.** Observed Risks and Estimated and Observed Risk Differences With Intensive vs Standard Blood Pressure Control by Tertiles of Estimated Benefit in SPRINT for Primary Outcome^a^

Estimated benefit tertile	No. of SPRINT participants		Primary outcome events, No. (%)			Absolute risk difference, intensive vs standard treatment group	
	Intensive (n = 3989)	Standard (n = 3929)	Intensive (n = 3989)	Standard (n = 3929)	Estimated (median)	Observed (95% CI)	Difference between observed and estimated
**Full set**							
High (−0.0504 to −0.0159)	1329	1310	513 (38.6)	526 (40.2)	−0.027	−0.013 (−0.054 to 0.028)	−0.014
Medium (−0.0159 to −0.00552)	1326	1313	181 (13.7)	221 (16.8)	−0.010	−0.032 (−0.06 to −0.003)	0.022
Low (−0.00552 to 0.000292)	1334	1305	71 (5.3)	81 (6.2)	−0.003	−0.008 (−0.025 to 0.010)	0.005
**Augmented reduced set^b^**							
High (−0.0512 to −0.0148)	1330	1317	501 (37.7)	526 (39.9)	−0.027	−0.006 (−0.047 to 0.034)	−0.02
Medium (−0.0148 to −0.00599)	1331	1315	185 (13.9)	215 (16.3)	−0.010	−0.038 (−0.067 to −0.010)	0.028
Low (−0.00599 to 0.00236)	1336	1311	80 (6.0)	91 (6.9)	−0.003	−0.007 (−0.026 to 0.011)	0.004
**Basic reduced set^c^**							
High (−0.0398 to −0.0194)	1338	1306	423 (31.6)	475 (36.4)	−0.026	−0.041 (−0.08 to −0.002)	0.015
Medium (−0.0194 to −0.0112)	1305	1339	219 (16.8)	225 (16.8)	−0.015	−0.002 (−0.032 to 0.028)	−0.014
Low (−0.0112 to 0.0024)	1351	1293	124 (9.2)	131 (10.1)	−0.007	−0.012 (−0.035 to 0.01)	0.005

Abbreviation: SPRINT, Systolic Blood Pressure Intervention Trial.

^a^ Primary outcome consists of probable dementia or amnestic mild cognitive impairment. Findings are derived from modified elastic-net Cox proportional hazards regression.

^b^ Includes most influential covariates based on prior knowledge regardless of whether they are readily available at the point of care.

^c^ Includes most influential covariates that are routinely available at point of care and/or can be easily obtained.

### Estimated Absolute Risks of Each Outcome

**Figure 2.**
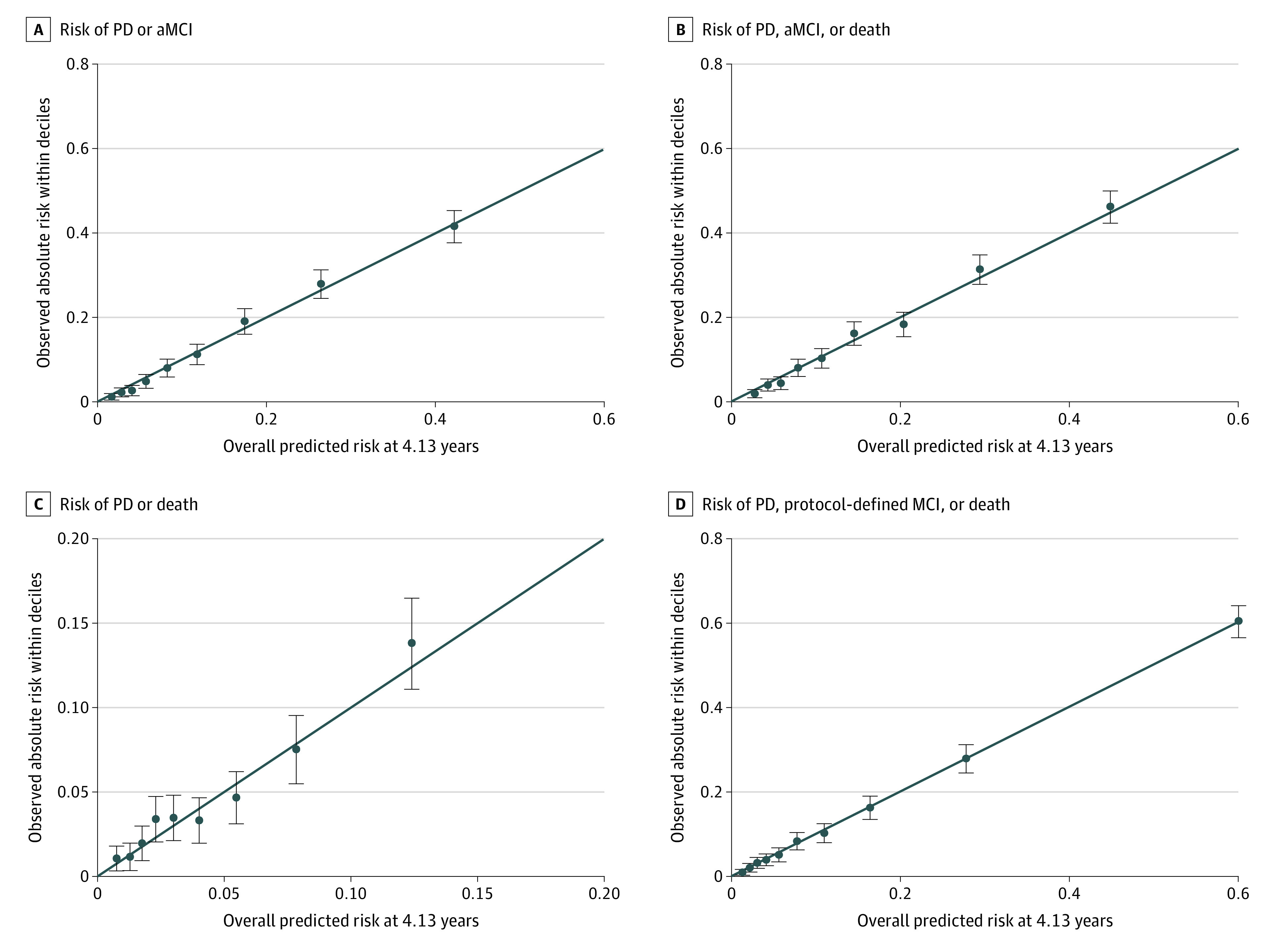
Observed Absolute Risks Within Deciles of Estimated Risk at 4.13 Years of Follow-up for the Primary Cognitive Outcome Calibration plots display the association between average risk estimated by elastic-net Cox proportional hazards regression model and observed risk estimated by Kaplan-Meier at 4.13 years for participants separated by decile of estimated risk. The solid blue diagonal lines show a perfect expected risk vs observed risk slope of 1. The points and whiskers present the observed absolute risk and 95% CIs across deciles of overall estimated risk. Calibration assesses how well elastic-net Cox proportional hazards regression model–estimated primary cognitive outcome event rates correspond to observed rates. A formal test of calibration was performed using the Greenwood-Nam-D'Agostino test. aMCI indicates amnestic mild cognitive impairment; PD, probable dementia.

The modified elastic-net Cox proportional hazards regression models estimating each of the outcomes using the full set performed well based on the C statistic, GND test, and MAE for the primary outcome of probable dementia and amnestic MCI (C statistic = 0.79; GND *P* = .23; MAE = 0.009); probable dementia, amnestic MCI, and death (C statistic = 0.79; GND *P* = .24; MAE = 0.011); probable dementia and death (C statistic = 0.82; GND *P* = .49; MAE = 0.007); and the composite of probable dementia, protocol-defined MCI, and death (C statistic = 0.81; GND *P* = .98; MAE = 0.003) (Figure 2 and eTable 6 in Supplement 2). Moreover, the modified elastic-net Cox proportional hazards models to estimate outcomes performed well with the reduced set and the basic reduced set (eFigures 2 and 3 in Supplement 2).

eFigure 4 in Supplement 2 shows the strength of each bivariate association for each covariate with the relevant outcome expressed as the standardized univariate HR. The baseline factors that were associated with higher risk for probable dementia and protocol-defined MCI were older age (HR per 1 SD, 1.87 [95% CI, 1.78-1.96]), being enrolled in Medicare (HR per 1 SD, 1.42 [95% CI, 1.35-1.49]), receiving insurance benefits through the Veterans Health Administration (HR per 1 SD, 1.15 [95% CI, 1.10-1.20]), and higher baseline serum creatinine level (HR per 1 SD, 1.24 [95% CI, 1.19-1.29]). Higher baseline cognitive assessment performance as measured with Montreal Cognitive Assessment, Logical Memory forms, serial 7s, and Digit Symbol Coding Test (HR per 1 SD, 0.43 [95% CI, 0.41-0.44]) and being employed (HR per 1 SD, 0.44 [95% CI, 0.42-0.46]) were associated with a lower risk of probable dementia or amnestic MCI. The patterns of associations were similar for the composites of probable dementia, amnestic MCI, or death and probable dementia and death as well as the composite of probable dementia, protocol-defined MCI, or death.

### Estimated Magnitude of Benefit With Intensive vs Standard Treatment

**Figure 3.**
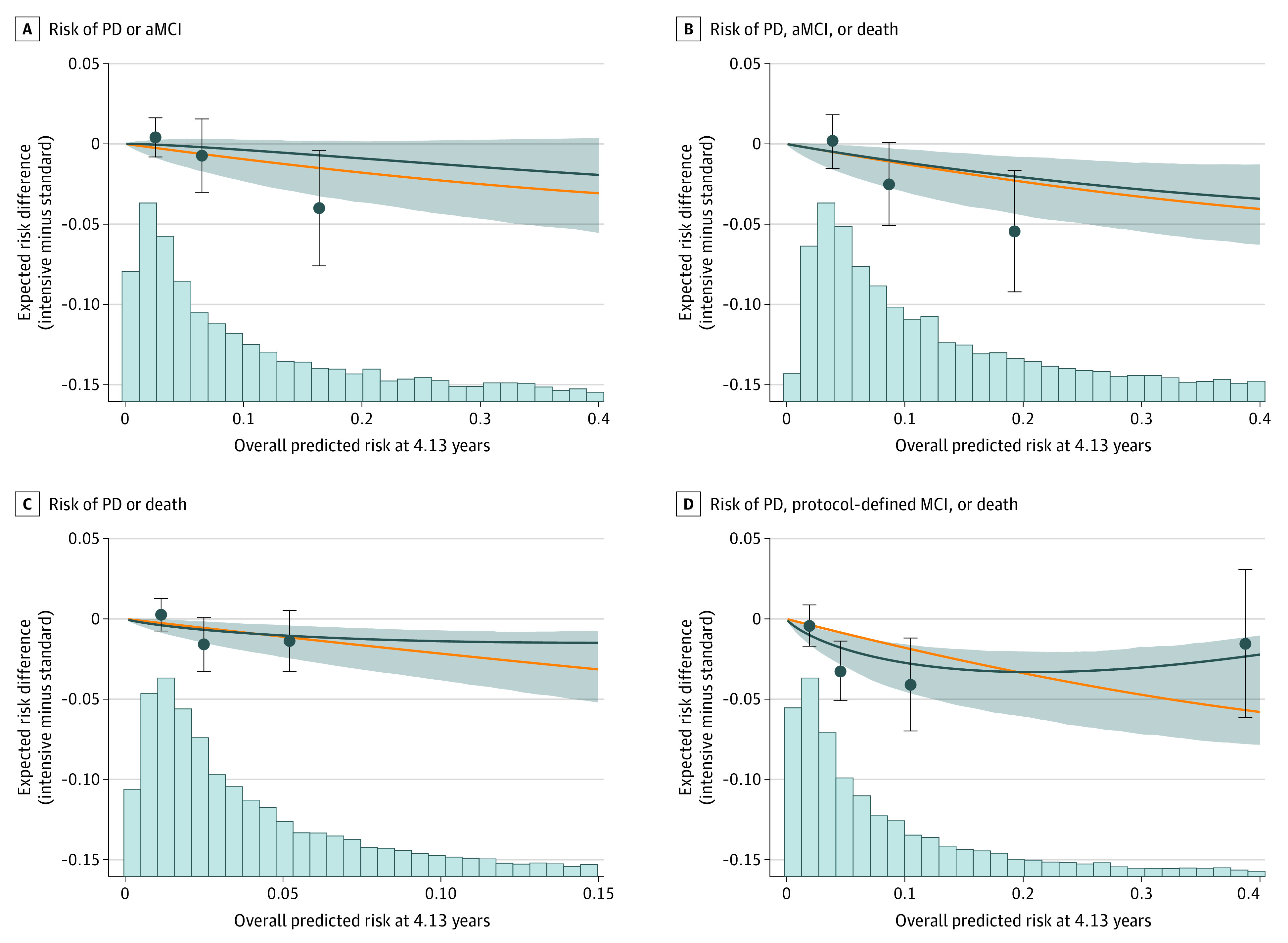
Estimated Magnitude of Benefit With Intensive vs Standard Systolic Blood Pressure Treatment Across a Range of Estimated Baseline Risk Solid orange lines represent direct risk magnification, where the absolute risk reduction is directly proportional to the baseline risk assuming a constant relative treatment effect on the hazard ratio scale. Solid blue lines represent the association between average elastic-net Cox proportional hazards regression model–estimated absolute risk difference for each outcome across the full range of baseline risk. The shaded gray areas represent the 95% CIs for the estimated risk difference. The histogram represents the distribution of baseline risk of each outcome in the Systolic Blood Pressure Intervention Trial population used in the current analysis. The blue circles represent the average observed risk difference for each outcome within quartiles of the Cox proportional hazards regression model–estimated absolute risk difference. The bars represent 95% CIs of the observed risk differences. aMCI indicates amnestic mild cognitive impairment; PD, probable dementia.

Risk magnification plots (Figure 3) show the association between the estimated treatment benefit (expressed as differences in risk between the treatment groups) and the estimated baseline risk for each cognitive outcome and for each feature set, separately. The models using the full covariate set are shown in Figure 3; those using the augmented reduced and basic reduced covariate sets are shown in eFigures 5 and 6 in Supplement 2. For the full covariate set, the risk amplification was approximately linear for the primary outcome of probable dementia and amnestic MCI (*P* = .69 for linearity). However, the association between baseline risk and effect of intensive vs standard SBP treatment on probable dementia or amnestic MCI; on probable dementia, amnestic MCI, or death; and on probable dementia or death tended to flatten at the highest level of baseline risk, with wide 95% CIs indicating substantial uncertainty. The risk amplification was approximately linear for the outcome of probable dementia or amnestic MCI or death using the full covariate set (*P* = .86 for linearity), augmented reduced set (*P* = .18 for linearity), and for the basic reduced set (*P* = .13 for linearity), with the association tending to flatten or even increase slightly, suggesting a less beneficial effect of the treatment at higher levels of baseline risk. The wide 95% CIs at high baseline risk reflect higher uncertainty in estimating the association between absolute risk and the risk difference at higher levels of baseline risk (eFigures 5 and 6 in Supplement 2).

The results of the hybrid risk- and effect-modeling analyses revealed that discrimination was not substantially improved from the full set, augmented reduced set, or basic reduced set in the primary analysis (eTable 6 in Supplement 2). Thus, it does not appear that estimation of the overall risk of our outcomes can be substantially improved by adding the interaction terms (treatment with each of age, sex, race, baseline MCI, and baseline CVD diagnosis subgroups).

## Discussion

Identifying baseline characteristics that project a higher magnitude of benefit within a clinical trial population is challenging yet important to advance personalized medicine.^5,33,34,35^ In this estimation modeling analysis of adjudicated cognitive outcomes in SPRINT, we found that higher baseline estimated risk of amnestic MCI or probable dementia was associated with greater absolute benefit from intensive (<120 mm Hg) compared with standard (<140 mm Hg) SBP treatment (Figure 3). These findings were consistent across all 3 covariate sets and after death was added to the composite outcome. We also found that the estimation ability of our models for amnestic MCI or probable dementia was not substantially reduced when using the augmented or basic covariate set, suggesting that simpler models perform similarly and may be preferred for implementation (eFigure 5 and 6 in Supplement 2). We found the strongest estimation factors for amnestic MCI or probable dementia were age, insurance status, kidney function, cognitive test results, and educational level (eFigure 2 in Supplement 2). Our results highlight the importance of prioritizing hypertension management of patients at increased risk of cognitive outcomes for intensive SBP treatment and are contrary to the belief of many that hypertension therapy is often limited value in older patients compared with middle-aged patients.

Hypertension is an important modifiable risk factor for CVD and dementia.^36,37,38^ Several cohort studies show that hypertension (SBP ≥140 mm Hg and SBP ≥130 mm Hg) is associated with higher risk of dementia.^38,39,40^ Additionally, a recent meta-analysis of 6 community-based prospective studies^37^ showed that antihypertensive medication use reduces the risk of dementia in patients with SBP of 140 mm Hg or higher or diastolic BP of 90 mm Hg or higher. These results are consistent with the SPRINT findings in which intensive SBP treatment significantly reduced the risk of a composite outcome of MCI or probable dementia (HR, 0.85 [95% CI, 0.74-0.97]).^1^ However, the double randomized Heart Outcomes Prevention Evaluation–3 Study^41^ showed that for older patients with intermediate cardiovascular risk with no prior CVD, long-term SBP lowering (median follow-up, 5.7 years) with candesartan cilexetil and hydrochlorothiazide did not affect cognitive or functional decline using a very limited cognitive battery insufficient to ascertain MCI or dementia. While some clinicians postulate that intensive BP control is too burdensome, SPRINT found that an average of only 1 additional medication is needed to achieve an SBP of less than 120 mm Hg, and serious adverse effects were not increased.^13^ Our results indicate that being older, having high serum creatinine levels, being enrolled in Medicare or Veterans Health Administration insurance, and having low baseline cognitive function most affected the association between intensive BP reduction and cognitive outcomes. These patients would benefit most from treatment intensification and should be targeted specifically for risk reduction. Additionally, we did not find evidence of effect modification on the relative hazard scale for prespecified subgroups (age, sex, race, baseline MCI [not adjudicated], and CVD diagnosis at baseline).

Overall, greater cognitive benefit was observed with intensive BP treatment in patients at greater risk of cognitive decline at baseline (Figure 3). Similar findings have been shown in other SPRINT outcomes, in which the greater the baseline CVD risk, the greater the CVD benefit of intensive SBP treatment.^3^ These data further support recommending intensive SBP treatment for patients who fulfill SPRINT eligibility criteria and have high estimated risk of CVD events or cognitive decline. Moreover, previous studies have shown that there is no increased risk for overall adverse effects by intensive treatment in SPRINT and that participants with the highest estimated CVD benefit from intensive treatment, and that although they are more likely to experience treatment-related adverse events, these were mild and transient.^2,3^ By maintaining cognitive function and avoiding CVD events, events that contributed significantly to loss of independence, duration and quality of life could be improved.^4^ This could be achieved with intensive BP control and is important to achieve as the population ages. In fact, over 12 million US adults are eligible to initiate or intensify antihypertensive treatment, and targeting populations based on baseline risk might be adventitious in limited resource settings.^42^

### Strengths and Limitations

These results should be interpreted within the context of the following strengths and limitations. Strengths include the use of complete SPRINT data and a randomized design. Additionally, cognitive outcomes were adjudicated by clinical experts in the care of persons with cognitive impairment and classified into meaningful clinical categories (no impairment, MCI, and probable dementia). Although we observed evidence of risk magnification, greater absolute risk reduction among patients with higher baseline risk of cognitive outcomes, the 95% CIs were wide among participants with high estimated absolute risk or an inverse association was observed due to small sample size (Figure 3). Given the limited existence of validated risk estimation models for cognitive impairment and dementia, we developed an internal risk model that we include in this report, and results may differ with external validation in other data sets or populations. We used several risk models based on covariates available in the clinical setting, although it is possible that we did not include a clinically informative variable if it was unmeasured in the SPRINT data (eg, family history of cognitive decline). Finally, we did not weigh the absolute benefits of intensive treatment on cognitive decline against the potential absolute harms (eg, serious adverse events) with intensive treatment.

## Conclusions

This secondary analysis of the SPRINT randomized clinical trial found that participants with higher baseline risk of MCI or dementia gained the greatest absolute benefit from intensive SBP treatment. The baseline clinical factors associated with risk of MCI or dementia include older age, worse kidney function, being enrolled in Medicare or Veterans Health Administration insurance, lower educational attainment, and low baseline cognitive function. If we are to prioritize intensifying antihypertensive treatment based on cognitive benefit, especially among patients who lack equitable access to health care and in resource-poor settings, we would recommend targeting patients at high risk of cognitive impairment initially. However, we had limited ability in identifying benefit in patients with high cognitive risk at baseline due to limited sample size.

## Notes

Supplement 1.
Trial Protocol

Supplement 2. **eMethods.** Study Data and Methods **eTable 1.** SPRINT Cognitive Battery Components **eTable 2.** Feature Sets Used in Estimation Models **eTable 3.** Observed Risks of the Benefit of the Primary Outcome (Total Time for PD, Amnestic MCI, or Death) and Estimated and Observed Risk Differences With Intensive vs Standard Blood Pressure Control by Tertiles of the Estimated Benefit in SPRINT for Secondary Outcome: Occurrence of Probable Dementia, Amnestic MCI, or Death **eTable 4.** Observed Risks of the Benefit of the Primary Outcome (Total Time for PD, Amnestic MCI, or Death) and Estimated and Observed Risk Differences With Intensive vs Standard Blood Pressure Control by Tertiles of the Estimated Benefit in SPRINT for Secondary Outcome: Probable Dementia or Death **eTable 5.** Observed Risks of the Benefit of the Primary Outcome (Total Time for PD, Amnestic MCI, or Death) and Estimated and Observed Risk Differences With Intensive vs Standard Blood Pressure Control by Tertiles of the Estimated Benefit in SPRINT for Secondary Outcome: Probable Dementia, Protocol-Defined MCI, or Death **eTable 6.** Comparing Estimation Performance of Models Used to Estimate Benefit for All Outcomes Using C Statistic **eFigure 1.** Timeline for Follow-up Cognitive Assessment in SPRINT **eFigure 2.** Calibration Plots Showing the Observed Absolute Risks Within Deciles of Estimated Risk at 4.13 Years Follow-up for the Primary Cognitive Outcome Using the Augmented Reduced Set **eFigure 3.** Calibration Plots Showing the Observed Absolute Risks Within Deciles of Estimated Risk at 4.13 Years Follow-up for the Primary Cognitive Outcome Using the Basic Reduced Set **eFigure 4.** Bar Graph Showing the Strength of Association of Each of the Factors in Each Feature Set With Cognitive Outcomes Benefit Expressed as the Standardized Univariate Hazard Ratios Associated With Each Outcome **eFigure 5.** Risk Magnification Plot Showing the Estimated Magnitude of Benefit With Intensive vs Standard Systolic BP Treatment Across a Range of Estimated Baseline Risk Using the Augmented Reduced Set **eFigure 6.** Risk Magnification Plot Showing the Estimated Magnitude of Benefit With Intensive vs Standard Systolic BP Treatment Across a Range of Estimated Baseline Risk Using the Basic Reduced Set

Supplement 3.
Data Sharing Statement
