# Supplementary material for: Identifying Patients for Intensive Blood Pressure Treatment Based on Cognitive Benefit: A Secondary Analysis of the SPRINT Randomized Clinical Trial
Source: JAMA Netw Open. 2023 May 19;6(5):e2314443. doi: 10.1001/jamanetworkopen.2023.14443 (PMC10199351; doi:10.1001/jamanetworkopen.2023.14443)
Supplement: Supplement 2. — eMethods. Study Data and Methods eTable 1. SPRINT Cognitive Battery Components eTable 2. Feature Sets Used in Estimation Models eTable 3. Observed Risks of the Benefit of the Primary Outcome (Total Time for PD, Amnestic MCI, or Death) and Estimated and Observed Risk Differences With Intensive vs Standard Blood Pressure Control by Tertiles of the Estimated Benefit in SPRINT for Secondary Outcome: Occurrence of Probable Dementia, Amnestic MCI, or Death eTable 4. Observed Risks of the Benefit of the Primary Outcome (Total Time for PD, Amnestic MCI, or Death) and Estimated and Observed Risk Differences With Intensive vs Standard Blood Pressure Control by Tertiles of the Estimated Benefit in SPRINT for Secondary Outcome: Probable Dementia or Death eTable 5. Observed Risks of the Benefit of the Primary Outcome (Total Time for PD, Amnestic MCI, or Death) and Estimated and Observed Risk Differences With Intensive vs Standard Blood Pressure Control by Tertiles of the Estimated Benefit in SPRINT for Secondary Outcome: Probable Dementia, Protocol-Defined MCI, or Death eTable 6. Comparing Estimation Performance of Models Used to Estimate Benefit for All Outcomes Using C Statistic eFigure 1. Timeline for Follow-up Cognitive Assessment in SPRINT eFigure 2. Calibration Plots Showing the Observed Absolute Risks Within Deciles of Estimated Risk at 4.13 Years Follow-up for the Primary Cognitive Outcome Using the Augmented Reduced Set eFigure 3. Calibration Plots Showing the Observed Absolute Risks Within Deciles of Estimated Risk at 4.13 Years Follow-up for the Primary Cognitive Outcome Using the Basic Reduced Set eFigure 4. Bar Graph Showing the Strength of Association of Each of the Factors in Each Feature Set With Cognitive Outcomes Benefit Expressed as the Standardized Univariate Hazard Ratios Associated With Each Outcome eFigure 5. Risk Magnification Plot Showing the Estimated Magnitude of Benefit With Intensive vs Standard Systolic BP Treatment Across a Range of Estimated Basel [file jamanetwopen-e2314443-s002.pdf]

Supplementary Online Content

Ghazi L, Shen J, Ying J, et al. Identifying patients for intensive blood pressure treatment based on cognitive benefit: a secondary analysis of the SPRINT randomized clinical trial. *JAMA Netw Open*. 2023;6(5):e2314443. doi:10.1001/jamanetworkopen.2023.14443

eMethods. Study Data and Methods
eTable 1. SPRINT Cognitive Battery Components
eTable 2. Feature Sets Used in Estimation Models
eTable 3. Observed Risks of the Benefit of the Primary Outcome (Total Time for PD, Amnestic MCI, or Death) and Estimated and Observed Risk Differences With Intensive vs Standard Blood Pressure Control by Tertiles of the Estimated Benefit in SPRINT for Secondary Outcome: Occurrence of Probable Dementia, Amnestic MCI, or Death
eTable 4. Observed Risks of the Benefit of the Primary Outcome (Total Time for PD, Amnestic MCI, or Death) and Estimated and Observed Risk Differences With Intensive vs Standard Blood Pressure Control by Tertiles of the Estimated Benefit in SPRINT for Secondary Outcome: Probable Dementia or Death
eTable 5. Observed Risks of the Benefit of the Primary Outcome (Total Time for PD, Amnestic MCI, or Death) and Estimated and Observed Risk Differences With Intensive vs Standard Blood Pressure Control by Tertiles of the Estimated Benefit in SPRINT for Secondary Outcome: Probable Dementia, Protocol-Defined MCI, or Death
eTable 6. Comparing Estimation Performance of Models Used to Estimate Benefit for All Outcomes Using C Statistic
eFigure 1. Timeline for Follow-up Cognitive Assessment in SPRINT
eFigure 2. Calibration Plots Showing the Observed Absolute Risks Within Deciles of Estimated Risk at 4.13 Years Follow-up for the Primary Cognitive Outcome Using the Augmented Reduced Set
eFigure 3. Calibration Plots Showing the Observed Absolute Risks Within Deciles of Estimated Risk at 4.13 Years Follow-up for the Primary Cognitive Outcome Using the Basic Reduced Set
eFigure 4. Bar Graph Showing the Strength of Association of Each of the Factors in Each Feature Set With Cognitive Outcomes Benefit Expressed as the Standardized Univariate Hazard Ratios Associated With Each Outcome
eFigure 5. Risk Magnification Plot Showing the Estimated Magnitude of Benefit With Intensive vs Standard Systolic BP Treatment Across a Range of Estimated Baseline Risk Using the Augmented Reduced Set
eFigure 6. Risk Magnification Plot Showing the Estimated Magnitude of Benefit With Intensive vs Standard Systolic BP Treatment Across a Range of Estimated Baseline Risk Using the Basic Reduced Set

This supplementary material has been provided by the authors to give readers additional information about their work.

© 2023 Ghazi L et al. *JAMA Network Open*.

eMethods. Study Data and Methods

Data Availability and Access

The authors take full responsibility for the data, the analyses and interpretation, and the conduct of the research. We had full access to all data, and we have the right to publish any and all data, separate and apart from the guidance of any sponsor. Limited versions of the data sets may be available through the Biologic Specimen and Data Repository Information Coordinating Center from the National Heart, Lung, and Blood Institute. Our analytic code is available to interested researchers from the corresponding author upon reasonable request.

© 2023 Ghazi L et al. *JAMA Network Open*.

The Systolic Blood Pressure Intervention Trial (SPRINT) Study Design and Intervention Detailed Summary

SPRINT enrolled the following participants: 1) Men and women age ≥ 50 years with systolic blood pressure (SBP) 130–180 mm Hg depending on the number of antihypertensive medications being taken; 2) Participants that had ≥ 1 high cardiovascular disease (CVD) risk conditions. High risk CVD conditions included the following: having a history of clinical or subclinical CVD other than stroke or having an estimated glomerular filtration rate (eGFR) of 20 to 59 mL/min/1.73m^2 using the four-variable Modification of Diet in Renal Disease equation; 3) Having a 10 year CVD risk ≥ 15% calculated using the Framingham Risk Score for general clinical practice, and/or being 75 years or older. Exclusion criteria included the following: diabetes, stroke history, heart failure, proteinuria defined as 1 or more grams/day, eGFR<20 mL/min/1.73 m^2. The protocol can be seen in the supplement as well for a full description.

<u>Intervention:</u> Patients were randomized to intensive SBP lowering (target <120 mmHg – intensive treatment group) or routine SBP measurement (target <140 mmHg – standard treatment group). Randomization was stratified according to clinical site. Both participants and the study personnel were aware of their treatment assignment, however adjudicators were blinded. Treatment algorithms are found below as well as in the complete SPRINT protocol at the end of the online supplement.

© 2023 Ghazi L et al. *JAMA Network Open*.

Figure A. Treatment algorithm for intensive treatment arm

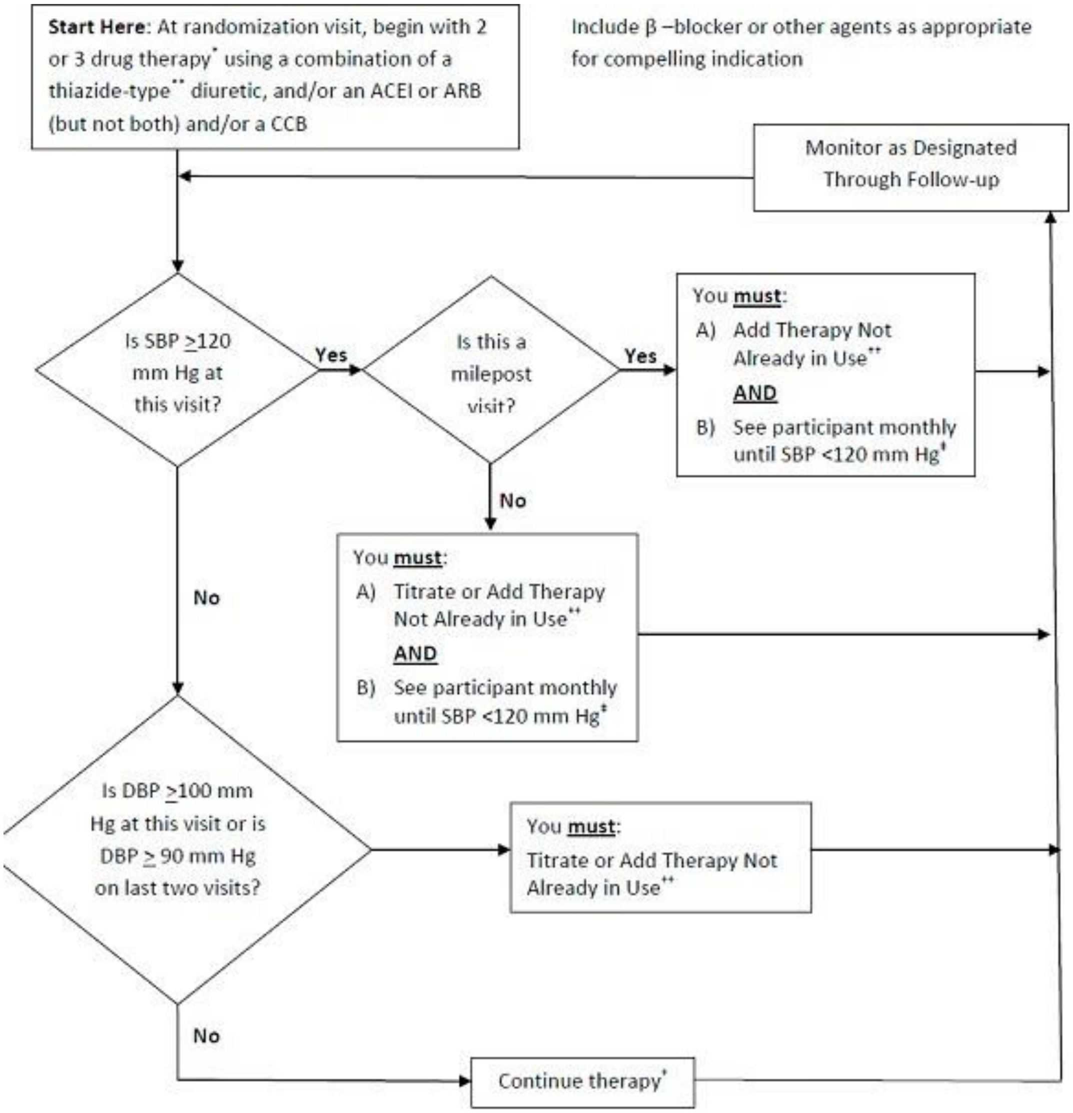

* May begin with a single agent for participants 75 years old or older with SBP < 140 on 0-1 meds at study entry. A second medication should be added at the 1 Month visit if participant is asymptomatic and SBP ≥ 130.

** May use loop diuretic for participants with advanced CKD

† Unless side effects warrant change in therapy

†† Consider consulting with the Clinical Center Network before adding a fifth anti-hypertensive medication

‡ Or until clinical decision made that therapy should not be increased further

© 2023 Ghazi L et al. *JAMA Network Open*.

Figure B. Treatment algorithm for the standard treatment arm

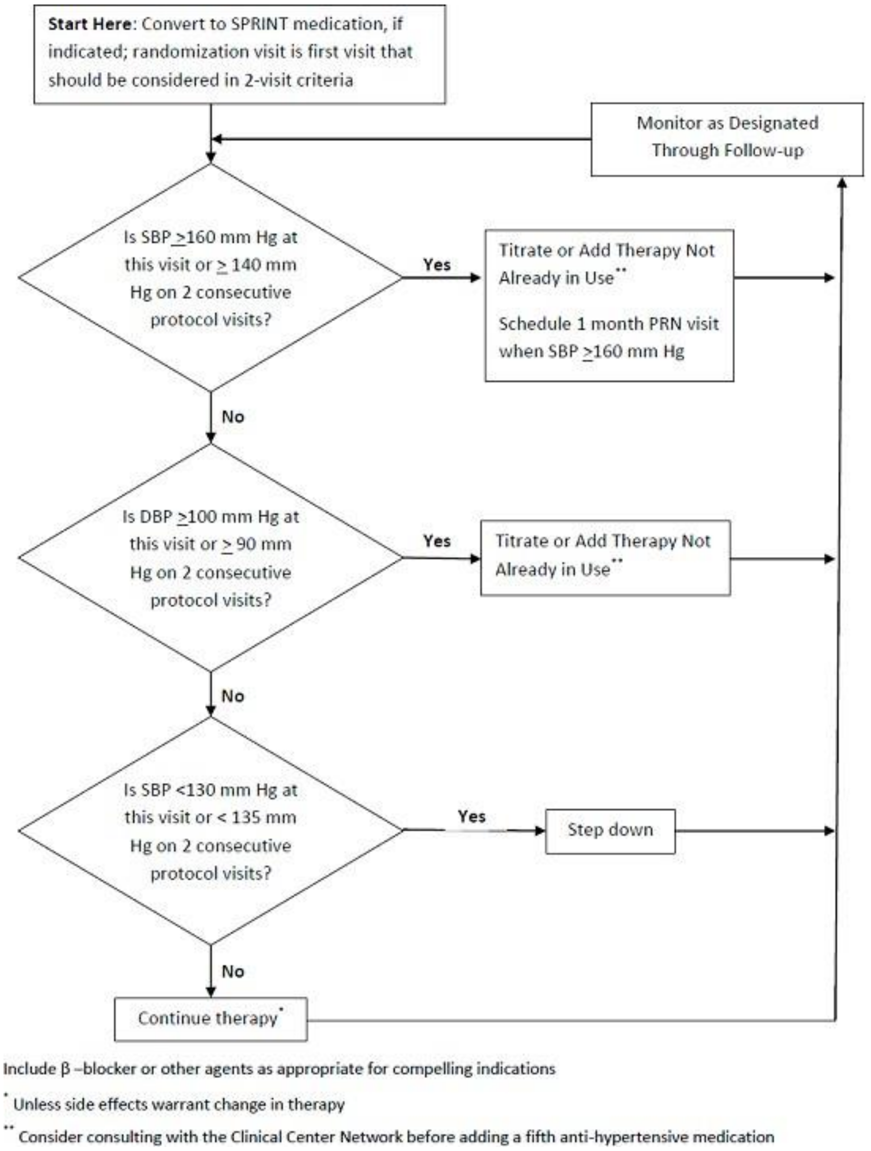

References:

1. Lewis CE, Fine LJ, Beddhu S, et al. Final Report of a Trial of Intensive versus Standard Blood-Pressure Control. N Engl J Med 2021;384:1921-1930.
2. Williamson JD, Pajewski NM, Auchus AP, et al. Effect of Intensive vs Standard Blood Pressure Control on Probable Dementia: A Randomized Clinical Trial. JAMA 2019;321:553-561.
3. Ambrosius WT, Sink KM, Foy CG, et al. The design and rationale of a multicenter clinical trial comparing two strategies for control of systolic blood pressure: the Systolic Blood Pressure Intervention Trial (SPRINT). Clin Trials 2014;11:532-546.
4. Systolic Blood Pressure Intervention Trial (SPRINT) Protocol Version 5.0 [online]. Available at: https://www.sprinttrial.org/public/Protocol_Current.pdf.

© 2023 Ghazi L et al. *JAMA Network Open*.

Cognitive Screening Test Administered

- MoCA for global cognitive function range: 0-30
- Logical Memory forms I and II subtests of the Wechsler Memory Scale for learning and memory range: 0-28 and 0-14
- A Functional Activities Questionnaire of 10 items to measure functional abilities was administered to a preidentified proxy for participants with a low score (White participants: <19 for those with <12 years of education or <21 for those with ≥12 years of education; Nonwhite participants: <17 for those with <12 years of education or <19 for those with ≥12 years of education) or a decrease of 5 or more points from a previous MoCA assessment. Score ranged between 0-30.

© 2023 Ghazi L et al. *JAMA Network Open*.

Estimation model development

The elastic net procedure is a generalization of the classic lasso and ridge regression methods and thus keeps the feature selection quality from the lasso penalty as well as the effectiveness of the ridge penalty.[1] For each cognitive outcome and covariate set, the standard elastic net Cox model was extended to relate the log hazard for the outcome to a baseline risk score (represents the average overall risk across both treatment groups), the treatment arm (intensive vs. standard), and an interaction term between these two, where the baseline risk score is defined as a linear combination of the set of covariates considered by the current model. Penalty terms were not applied to the coefficient of the treatment arm or its interaction with the baseline risk score. After appropriate transformation, the model accounts for a potential smooth non-linear relationship between the magnitude of the treatment effect on the difference in the risk of the outcome between the treatment groups and the estimated baseline absolute risk, while retaining the assumption that the estimated treatment effect must be homogeneous/constant as the estimated baseline risk score approaches 0.

For all outcomes and covariate sets, we used the resulting modified elastic net regression model to estimate overall absolute risk at 4.13 years, the median follow up time in SPRINT for cognitive outcomes in the current analysis. To evaluate the performance of different models, formal measures of model discrimination and model calibration were considered (1). Discrimination characterizes the model's ability to accurately rank patients' absolute risk of the event from low to high. As a frequently utilized model discrimination statistic, the concordance statistic (C-statistic) measures the proportion of subject pairs that have agreement on the order of estimated risk and observed event free survival time among all ordered pairs (2). It thus

© 2023 Ghazi L et al. *JAMA Network Open*.

respectively provides a summary of accuracy at a specified time and an overall measure of estimation accuracy. In this study, we used cross-validated c-statistics to assess the model discrimination, where 5-fold cross-validation was employed in absolute risk estimations to avoid over-fitting.

Model calibration refers to how accurately the model's estimations match actual event rates. Here, we used the Greenwood–Nam–D'Agostino (GND) test[4,5] to assess the calibration of our risk estimation models. We stratified the study cohort into 10 groups using deciles of the estimated risk scores from a given model and compared the mean absolute risk estimated by the model versus risk estimated by the Kaplan-Meier method for each group. The GND test was then performed. In addition, the mean absolute error (MAE) for the comparison across all 10 groups was calculated as another metric to assess how close the estimated absolute risks are to the observed values.

Hybrid risk modeling and effect modeling: In order to assess the existence of additional heterogeneity in treatment benefits which is not explained by variation in baseline risk, we considered a sensitivity analysis with a hybrid risk modeling and effect modeling approach. In this approach we added extra interaction terms between treatment assignment and certain prespecified subgroups to the modified elastic net Cox regression models. These subgroup variables were prespecified based on clinical knowledge, which included age <75 vs. ≥75 years, female vs. male sex, race (black vs. non-black), with and without baseline MCI, with and without baseline CVD. Risk modeling and effect modeling are two distinct approaches in the analysis of heterogeneity of treatment effects (HTE).(3) Risk modeling focuses on the dependency of treatment effect on the absolute risk in the control arm, an observable proxy for outcome risk. Because the absolute risk is often the most clinically important effect measure, risk modeling is

© 2023 Ghazi L et al. *JAMA Network Open*.

considered to be a good choice in identifying “clinically important HTE,” which is evaluated on the absolute risk difference scale. On the other hand, effect modeling usually direct targets a regression model on entire dataset with inclusion of risk estimators and a treatment assignment variable, as well as the treatment interaction terms. This approach can be vulnerable to some of the same problems that undermine conventional subgroup analysis, including low statistical power, multiplicity, and limited prior knowledge about important effect modifiers. (4)

References:
1. Austin, P. C., & Steyerberg, E. W. (2012). Interpreting the concordance statistic of a logistic regression model: relation to the variance and odds ratio of a continuous explanatory variable. *BMC medical research methodology*, *12*, 1-8.
2. Pencina, M. J., & D’Agostino, R. B. (2015). Evaluating discrimination of risk prediction models: the C statistic. *Jama*, *314*(10), 1063-1064.
3. Kent, D. M., Paulus, J. K., Van Klaveren, D., D'Agostino, R., Goodman, S., Hayward, R., ... & Steyerberg, E. W. (2020). The predictive approaches to treatment effect heterogeneity (PATH) statement. *Annals of internal medicine*, *172*(1), 35-45.
4. Kent, D. M., Steyerberg, E., & van Klaveren, D. (2018). Personalized evidence based medicine: predictive approaches to heterogeneous treatment effects. *Bmj*, *363*.

© 2023 Ghazi L et al. *JAMA Network Open*.

eTable 1. SPRINT Cognitive Battery Components

	Components of In-Person Cognitive Screening Battery	Components of In-Person Cognitive Extended Battery	Components of Telephone Cognitive Battery
Global Functioning	Montreal Cognitive Assessment		Modified Telephone Interview for Cognitive Status
Executive Function	Digit Symbol Coding Test		
Speed of Processing		Trail Making Test Parts A and B	Oral Trail Making Test Parts A and B
Learning and Memory	Logical Memory I	Hopkins Verbal Learning Test-Revised	
Visual-Spatial Memory		Modified Rey-Osterreith Complex Figure	
Working Memory and Attention		Digit Span Forward and Backward	
Verbal Fluency		Category Fluency-Animals	Category Fluency-Animals
Language and Naming		Boston Naming Test	

© 2023 Ghazi L et al. *JAMA Network Open*.

eTable 2. Feature Sets Used in Estimation Models

	**Full Set**	**Augmented Reduced Set**	**Basic Reduced Set**
Demographics			
Age	+	+	+
Female	+	+	+
Race/Ethnicity			
Black	+	+	+
Non-Black	+	+	+
Social and Behavioral			
Lives with others	+	+	
Has private insurance	+	+	+
Current smoker	+	+	+
Former smoker	+	+	+
Never smoker	+	+	+
Employed	+		+
Engages in vigorous physical activity (≥15minutes/day)	+	+	+
Alcohol consumption (number of drinks/day)	+	+	+
Education	+	+	+
Less than high school	+	+	+
High school graduate only	+	+	+
Post high school graduate	+	+	+
College graduate or greater	+	+	+
Health insurance status			
Uninsured	+	+	+
Medicare	+	+	+
Medicaid	+	+	+
Private	+	+	+
VA	+	+	+

© 2023 Ghazi L et al. *JAMA Network Open*.

	Full Set	Augmented Reduced Set	Basic Reduced Set
Usual Source of Care			
Doctors office/outpatient clinic	+		+
Community healthcare facility/other	+		+
No usual source of care	+		+
Medical History			
Clinical CVD	+	+	+
Left ventricular hypertrophy	+	+	+
Dizziness when standing	+	+	+
History of coronary revascularization	+	+	+
History of depression (self report)	+	+	+
Baseline cognitive assessments			
Montreal Cognitive Assessment	+	+	
Logical Memory form II	+	+	
Digit Symbol Coding Test	+	+	
Serial 7's			+
Clinical/Laboratory Measurements			
Systolic BP	+	+	+
Diastolic BP	+	+	+
Resting heart rate	+	+	+
Serum potassium	+	+	+
Serum creatinine	+	+	+
Albumin to creatinine ratio	+	+	+
Total cholesterol	+	+	+
HDL cholesterol	+	+	+
Triglycerides	+	+	+
Body mass index	+	+	+
Serum glucose	+	+	+
Medication Use			
Aspirin	+		+

© 2023 Ghazi L et al. *JAMA Network Open*.

	Full Set	Augmented Reduced Set	Basic Reduced Set
Medication Use			
Statin	+	+	+
NSAID	+		+
Benzodiazepines	+	+	+
Anticholinergics	+	+	+
Antidepressants	+	+	+
Number of non-antihypertensive medications	+		+
Number of antihypertensive medications	+	+	+
ARB	+		+
ACE-I	+		+
DHP CCB	+		+
Non-DHP CCB	+		+
Thiazide diuretic	+		+
Loop diuretic	+		+
Beta-blocker	+		+
Alpha-blocker	+		+
Other antihypertensive medication	+		+

ACEI: angiotensin-converting enzyme inhibitor; ARB: angiotensin-II receptor blocker; CCB: calcium channel blocker; CVD: cardiovascular disease; BP: blood pressure; HDL: high-density lipoprotein; NSAID: non-steroidal anti-inflammatory drug; SPRINT: Systolic Blood Pressure Intervention Trial

© 2023 Ghazi L et al. *JAMA Network Open*.

eTable 3. Observed Risks of the Benefit of the Primary Outcome (Total Time for PD, Amnestic MCI, or Death) and Estimated and Observed Risk Differences With Intensive vs Standard Blood Pressure Control by Tertiles of the Estimated Benefit in SPRINT for Secondary Outcome: Occurrence of Probable Dementia, Amnestic MCI, or Death*

**Estimated Benefit Tertile**	**Number of SPRINT Participants**		**Number of Primary Outcome Events* (%)**		**Absolute risk difference with intensive vs. standard**		
	**Intensive** (N=3,989)	**Standard** (N=3,929)	**Intensive** (N=3,989)	**Standard** (N=3,929)	**Estimated (median)**	**Observed (95% CI)**	**Difference**
**A. Full set**							
High (-0.0525,-0.0237)	1346	1293	472(35.1%)	480(37.1%)	-0.035	-0.017 (-0.056, 0.022)	-0.018
Medium (-0.0237,-0.0124)	1295	1344	208(16.1%)	302(22.5%)	-0.017	-0.062 (-0.093, -0.032)	0.045
Low (-0.0124,0.00398)	1348	1291	174(12.9%)	172(13.3%)	-0.008	-0.002 (-0.028, 0.024)	-0.006
**B. Augmented reduced set (include most influential covariates based on prior knowledge regardless if they are readily available at the point of care)**							
High (-0.0646,-0.0244)	1337	1310	479(35.8%)	512(39.1%)	-0.035	-0.015 (-0.054, 0.024)	-0.02
Medium (-0.0244,-0.0128)	1321	1325	212(16%)	262(19.8%)	-0.018	-0.052 (-0.083, -0.022)	0.034
Low (-0.0128,0.0094)	1339	1308	164(12.2%)	184(14.1%)	-0.008	-0.014 (-0.04, 0.012)	0.006
**C. Basic reduced set (includes covariates that are routinely available at point of care and/or can be easily obtained)**							
High (-0.0516,-0.0268)	1320	1324	445(33.7%)	498(37.6%)	-0.034	-0.033 (-0.072, 0.006)	-0.002
Medium (-0.0268,-0.0171)	1308	1336	243(18.6%)	299(22.4%)	-0.022	-0.037 (-0.069, -0.006)	0.015
Low (-0.0171,0.00432)	1366	1278	167(12.2%)	160(12.5%)	-0.011	-0.006 (-0.031, 0.02)	-0.006

*Findings derived from modified elastic net Cox regression
CI: confidence interval; PD: probable dementia, MCI: mild cognitive impairment; SPRINT: Systolic Blood Pressure Intervention Trial

© 2023 Ghazi L et al. *JAMA Network Open*.

eTable 4. Observed Risks of the Benefit of the Primary Outcome (Total Time for PD, Amnestic MCI, or Death) and Estimated and Observed Risk Differences With Intensive vs Standard Blood Pressure Control by Tertiles of the Estimated Benefit in SPRINT for Secondary Outcome: Probable Dementia or Death*

Estimated Benefit Tertile	Number of SPRINT Participants		Number of Primary Outcome Events* (%)		Absolute risk difference with intensive vs. standard		
	**Intensive** (N=3,989)	**Standard** (N=3,929)	**Intensive** (N=3,989)	**Standard** (N=3,929)	**Estimated (median)**	**Observed (95% CI)**	**Difference**
**A. Full set**							
High (-0.0485,-0.0144)	1347	1293	173(12.8%)	189(14.6%)	-0.025	-0.017 (-0.045, 0.012)	-0.009
Medium (-0.0144,-0.00751)	1295	1344	58(4.5%)	86(6.4%)	-0.011	-0.013 (-0.031, 0.005)	0.002
Low (-0.00751,0.0322)	1347	1292	25(1.9%)	39(3%)	-0.005	-0.012 (-0.024, 0.001)	0.007
**B. Augmented reduced set (include most influential covariates based on prior knowledge regardless if they are readily available at the point of care)**							
High (-0.0789,-0.0139)	1332	1315	180(13.5%)	218(16.6%)	-0.026	-0.026 (-0.056, 0.003)	0.001
Medium (-0.0139,-0.00686)	1316	1331	43(3.3%)	68(5.1%)	-0.01	-0.018 (-0.034, -0.002)	0.009
Low (-0.00686,0.0363)	1349	1298	33(2.4%)	28(2.2%)	-0.004	0.006 (-0.006, 0.018)	-0.011
**C. Basic reduced set (includes covariates that are routinely available at point of care and/or can be easily obtained)**							
High (-0.0444,-0.0151)	1342	1303	165(12.3%)	191(14.7%)	-0.022	-0.023 (-0.052, 0.005)	0.001
Medium (-0.0151,-0.00832)	1329	1315	45(3.4%)	80(6.1%)	-0.011	-0.024 (-0.041, -0.007)	0.013
Low (-0.00832,0.0458)	1323	1321	46(3.5%)	43(3.3%)	-0.005	0.006 (-0.008, 0.02)	-0.011

*Findings derived from modified elastic net Cox regression
CI: confidence interval; PD: probable dementia, MCI: mild cognitive impairment; SPRINT: Systolic Blood Pressure Intervention Trial

© 2023 Ghazi L et al. *JAMA Network Open*.

eTable 5. Observed Risks of the Benefit of the Primary Outcome (Total Time for PD, Amnestic MCI, or Death) and Estimated and Observed Risk Differences With Intensive vs Standard Blood Pressure Control by Tertiles of the Estimated Benefit in SPRINT for Secondary Outcome: Probable Dementia, Protocol-Defined MCI, or Death*

**Estimated Benefit Tertile**	**Number of SPRINT Participants**		**Number of Primary Outcome Events* (%)**		**Absolute risk difference with intensive vs. standard**		
	**Intensive** (N=3,989)	**Standard** (N=3,929)	**Intensive** (N=3,989)	**Standard** (N=3,929)	**Estimated (median)**	**Observed (95% CI)**	**Difference**
**A. Full set**							
High (-0.0436,-0.0274)	1320	1320	205(15.5%)	251(19%)	-0.033	-0.034 (-0.065, -0.003)	0.001
Medium (-0.0274,-0.0155)	1322	1317	111(8.4%)	140(10.6%)	-0.021	-0.023 (-0.047, 0)	0.002
Low (-0.0155,0.0644)	1347	1292	166(12.3%)	183(14.2%)	-0.006	-0.017 (-0.044, 0.009)	0.012
**B. Augmented reduced set (include most influential covariates based on prior knowledge regardless if they are readily available at the point of care)**							
High (-0.0447,-0.0263)	1307	1340	213(16.3%)	261(19.5%)	-0.033	-0.031 (-0.062, 0.001)	-0.002
Medium (-0.0263,-0.0151)	1337	1310	95(7.1%)	134(10.2%)	-0.02	-0.033 (-0.056, -0.01)	0.013
Low (-0.0151,0.0636)	1353	1294	174(12.9%)	180(13.9%)	-0.006	-0.008 (-0.035, 0.018)	0.002
**C. Basic reduced set (includes covariates that are routinely available at point of care and/or can be easily obtained)**							
High (-0.0511,-0.0259)	1331	1314	213(16%)	244(18.6%)	-0.035	-0.023 (-0.054, 0.008)	-0.013
Medium (-0.0259,-0.0167)	1331	1313	101(7.6%)	136(10.4%)	-0.021	-0.034 (-0.058, -0.011)	0.013
Low (-0.0167,0.112)	1332	1312	168(12.6%)	195(14.9%)	-0.006	-0.018 (-0.045, 0.008)	0.012

*Findings derived from modified elastic net Cox regression
CI: confidence interval; PD: probable dementia, MCI: mild cognitive impairment; SPRINT: Systolic Blood Pressure Intervention Trial

© 2023 Ghazi L et al. *JAMA Network Open*.

eTable 6. Comparing Estimation Performance of Models Used to Estimate Benefit for All Outcomes Using C Statistic

	**Primary Outcome (PD or amnestic MCI)**			**Secondary Outcome (PD, amnestic MCI or death)**			**Secondary Outcome (PD or death)**			**Secondary Outcome (PD, protocol defined MCI or death)**		
	**C-Statistic**			**C-Statistic**			**C-Statistic**			**C-Statistic**		
**Modeling Approach**	A. Full set	B. Augmented reduced set	C. Basic reduced set	A. Full set	B. Augmented reduced set	C. Basic reduced set	A. Full set	B. Augmented reduced set	C. Basic reduced set	A. Full set	B. Augmented reduced set	C. Basic reduced set
Modified elastic net cox regression	0.7874	0.8027	0.7741	0.7926	0.7956	0.7631	0.8193	0.8255	0.8107	0.8129	0.8144	0.7874
Hybrid Risk- and Effect-Modeling	0.8036	0.7891	0.7792	0.7916	0.7792	0.7796	0.8183	0.8225	0.815	0.8127	0.8134	0.8036

PD: probable dementia, MCI: mild cognitive impairment
Augmented reduced set: includes the most influential covariates based on prior knowledge regardless if they are readily available at the point of care
Basic reduced set: includes covariates that are routinely available at point of care and/or can be easily obtained

© 2023 Ghazi L et al. *JAMA Network Open*.

eFigure 1. Timeline for Follow-up Cognitive Assessment in SPRINT

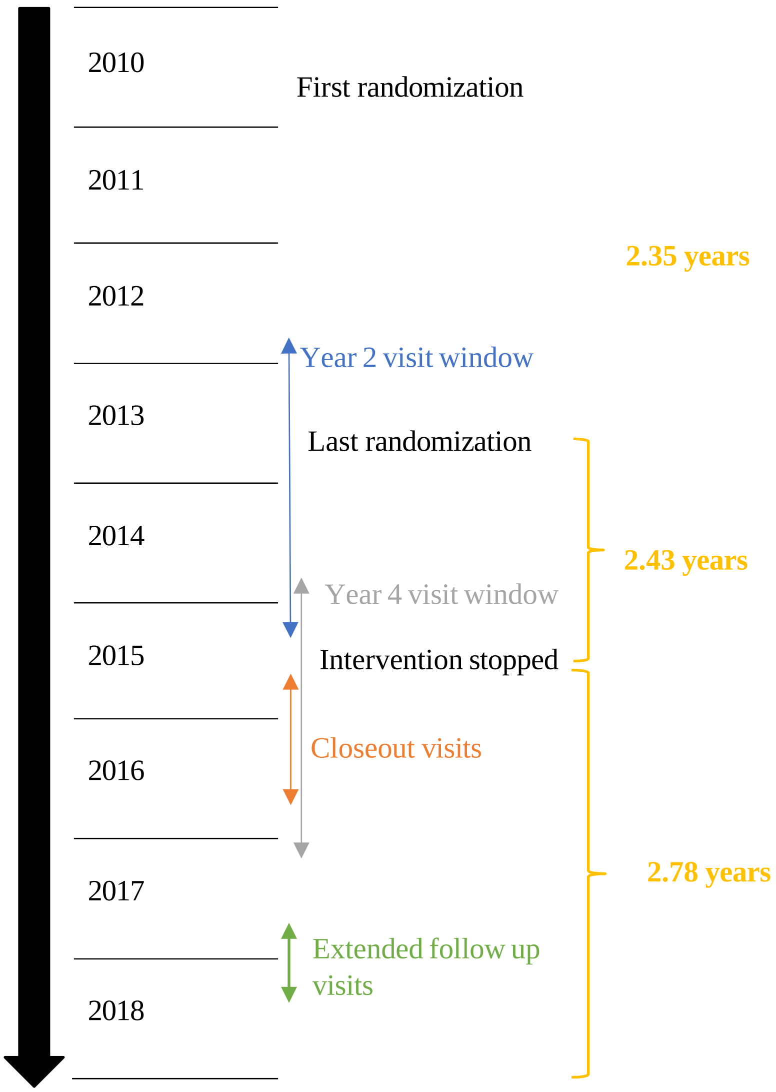

© 2023 Ghazi L et al. *JAMA Network Open*.

eFigure 2. Calibration Plots Showing the Observed Absolute Risks Within Deciles of Estimated Risk at 4.13 Years Follow-up for the Primary Cognitive Outcome Using the Augmented Reduced Set

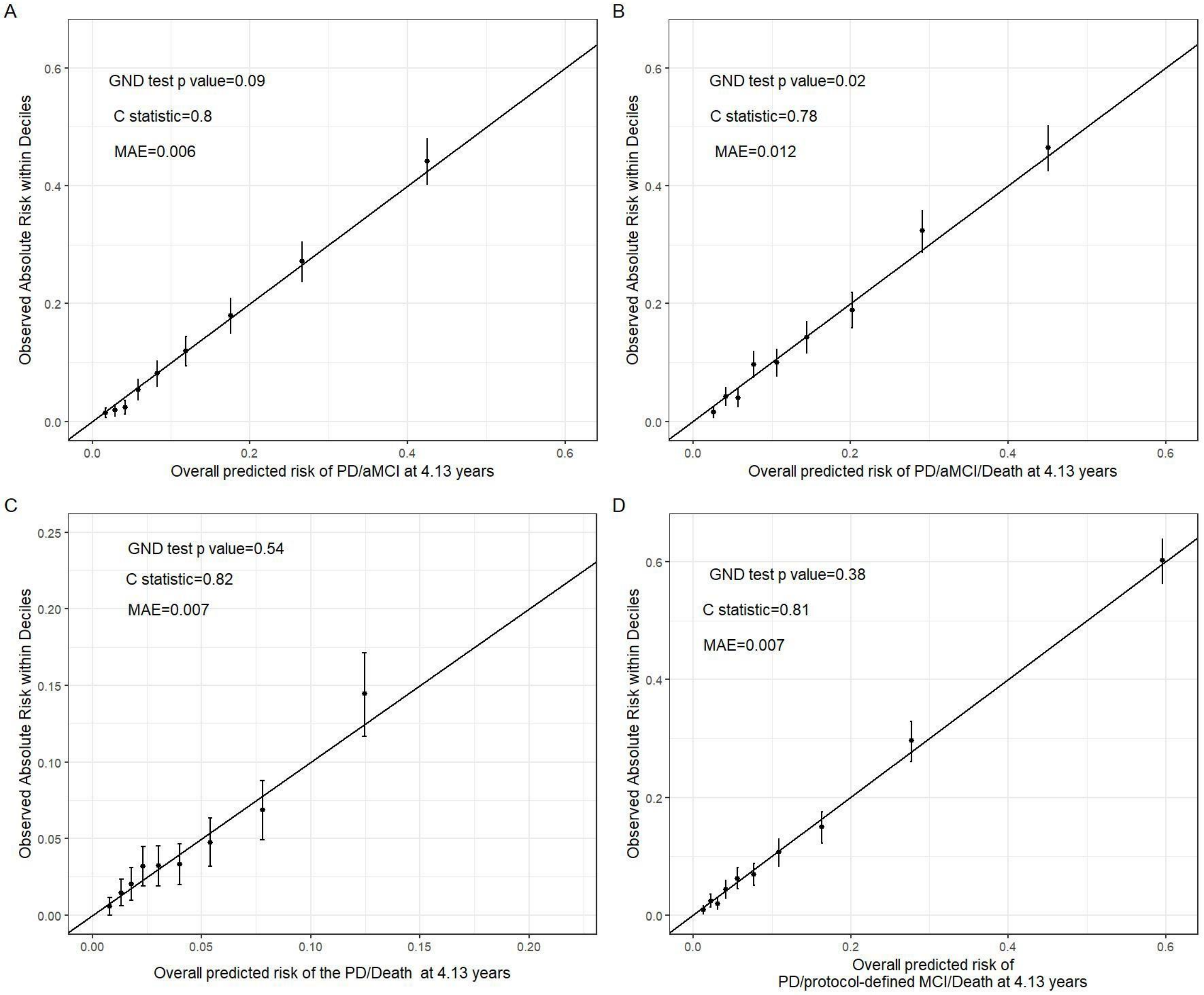

Shown are calibration plots displaying the relationship between average estimated risk estimated by elastic net Cox-model- and observed risk estimated by Kaplan-Meier at 4.13 years for subjects separated by decile of estimated risk. The solid black diagonal lines show a perfect expected risk versus observed risk slope of 1. The points and whiskers present the observed absolute risk and 95% CI across deciles of overall estimated risk. Panel A corresponds to risk of Probable Dementia (PD) or amnestic Mild Cognitive Impairment (aMCI); Panel B corresponds to the risk of PD or aMCI or death; Panel C corresponds to risk of PD or Death; Panel D corresponds to the risk of PD or protocol defined MCI or death. Calibration assesses how well elastic net Cox-model-estimated primary cognitive outcome event rates correspond to observed rates. A formal test of calibration was performed using and the Greenwood–Nam–D'Agostino (GND) test. Mean absolute error (MAE) is mean absolute error.

© 2023 Ghazi L et al. *JAMA Network Open*.

eFigure 3. Calibration Plots Showing the Observed Absolute Risks Within Deciles of Estimated Risk at 4.13 Years Follow-up for the Primary Cognitive Outcome Using the Basic Reduced Set

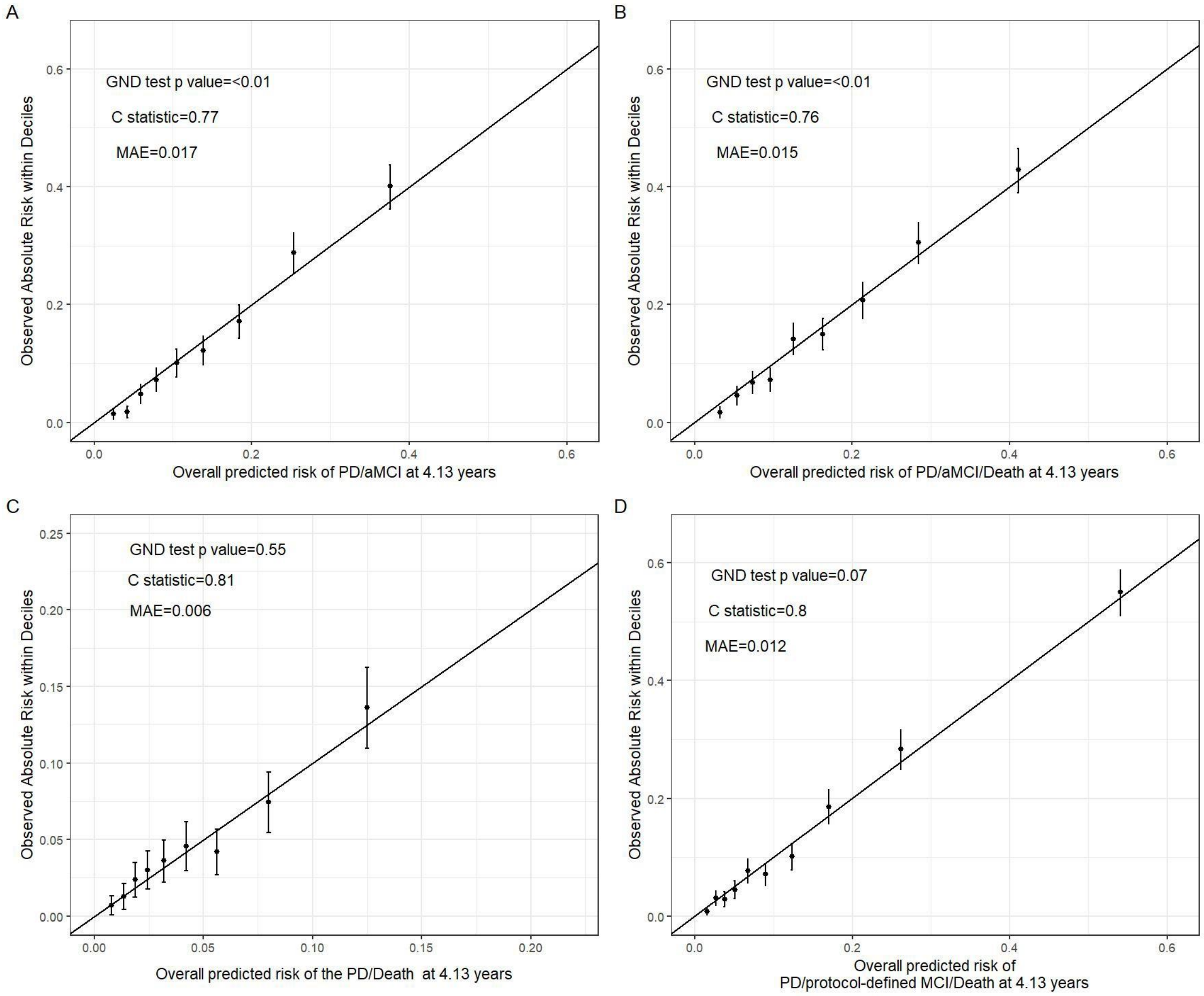

Shown are calibration plots displaying the relationship between average estimated risk estimated by elastic net Cox-model- and observed risk estimated by Kaplan-Meier at 4.13 years for subjects separated by decile of estimated risk. The solid black diagonal lines show a perfect expected risk versus observed risk slope of 1. The points and whiskers present the observed absolute risk and 95% CI across deciles of overall estimated risk. Panel A corresponds to risk of Probable Dementia (PD) or amnestic Mild Cognitive Impairment (aMCI); Panel B corresponds to the risk of PD or aMCI or death; Panel C corresponds to risk of PD or Death; Panel D corresponds to the risk of PD or protocol defined MCI or death. Calibration assesses how well elastic net Cox-model-estimated primary cognitive outcome event rates correspond to observed rates. A formal test of calibration was performed using and the Greenwood–Nam–D'Agostino (GND) test. Mean absolute error (MAE) is mean absolute error.

© 2023 Ghazi L et al. *JAMA Network Open*.

eFigure 4. Bar Graph Showing the Strength of Association of Each of the Factors in Each Feature Set With Cognitive Outcomes Benefit Expressed as the Standardized Univariate Hazard Ratios Associated With Each Outcome

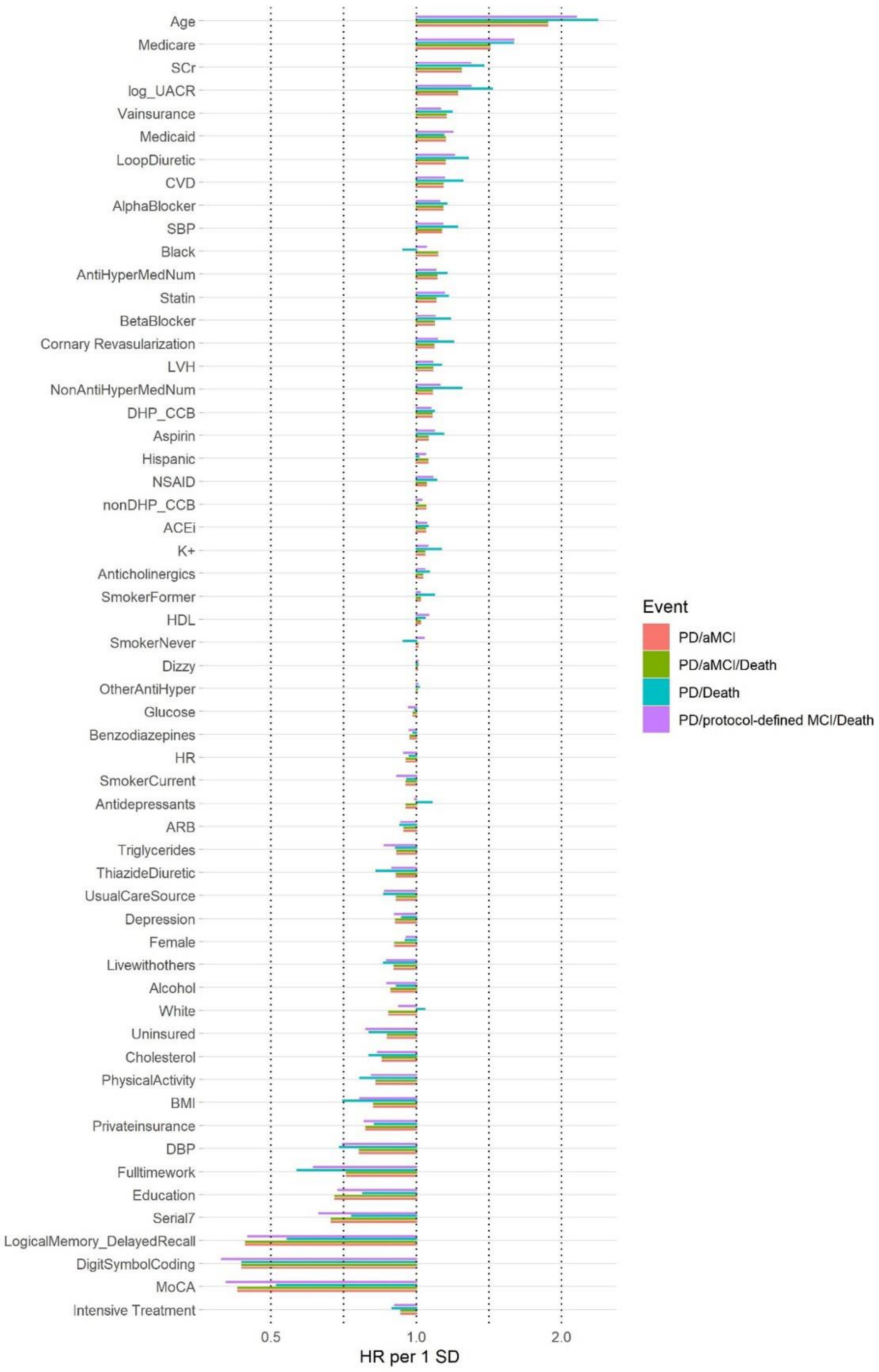

© 2023 Ghazi L et al. *JAMA Network Open*.

Bar graph showing the strength of association of each of the factors in each feature set with cognitive outcomes benefit expressed as the standardized univariate hazard ratios associated with each outcome
Each horizontal bar describes the bivariate standardized hazard ratio for each outcome (Probable Dementia (PD) or amnestic mild cognitive impairment (aMCI) in orange; PD or aMCI or death in green, and PD or death in blue, and PD or protocol defined MCI in purple associated with each baseline variable described on the y-axis. SD: standard deviation

SCr: creatinine at baseline; log_UACR: log urine albumin to creatinine ratio; Vainsurance: Veterans Affairs Insurance; CVD: cardiovascular disease; SBP: systolic blood pressure; ANtiHyperMedNum: number of antihypertensive medications;; LVH: Left ventricular hypertrophy; DHP_CCB: dihydropyridine calcium channel blockers; NSAID: non-steroidal anti-inflammatory; nonDHP_CCB: non dihydropyridine calcium channel blockers; ACEi: angiotensin receptor blocker inhibitors; K+: potassium: HDL: high density lipoprotein; HR: hear rate; ARB: angiotensin receptor blocker; BMI: body mass index; DBP: diastolic blood pressure

© 2023 Ghazi L et al. *JAMA Network Open*.

eFigure 5. Risk Magnification Plot Showing the Estimated Magnitude of Benefit With Intensive vs Standard Systolic BP Treatment Across a Range of Estimated Baseline Risk Using the Augmented Reduced Set

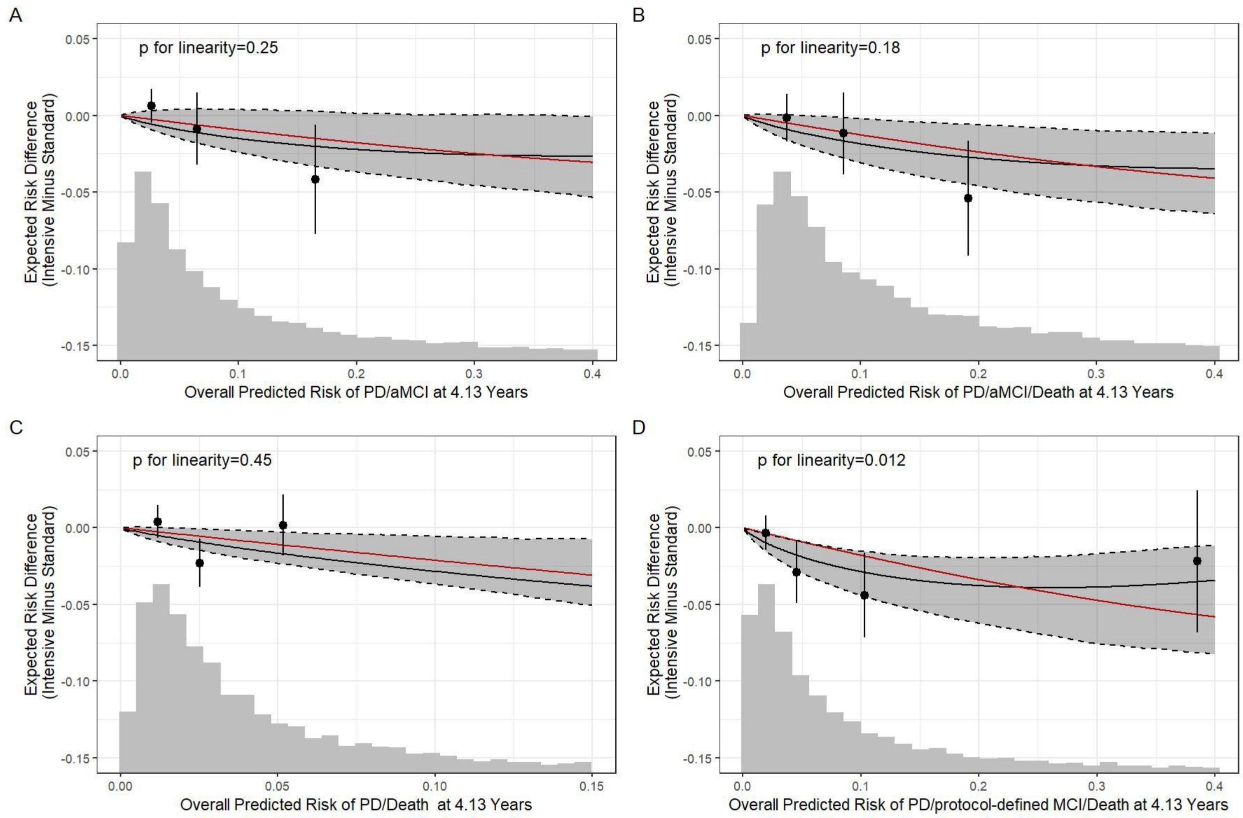

The solid red line represents direct risk magnification, where the absolute risk reduction is directly proportional to the baseline risk assuming a constant relative treatment effect on the hazard ratio scale. The solid black line represents the relationship between average elastic net Cox-model-estimated absolute risk difference for each outcome across the full range of baseline risk. The dashed black line and shaded grey areas represent the 95% confidence bands for the estimated risk difference. The histogram represents the distribution of baseline risk of each outcome in the SPRINT population used in the current analysis. The black circles represent the average observed risk difference for each outcome within quartiles of the Cox-model-estimated absolute risk difference. The bars represent 95% confidence limits of the observed risk differences. Panel A corresponds to risk of probable dementia (PD) or amnestic Mild Cognitive Impairment (aMCI); Panel B corresponds to the risk of PD or aMCI or death; Panel C corresponds to the risk of PD or death; and Panel D corresponds to the risk of PD or protocol defined MCI or death

© 2023 Ghazi L et al. *JAMA Network Open*.

eFigure 6. Risk Magnification Plot Showing the Estimated Magnitude of Benefit With Intensive vs Standard Systolic BP Treatment Across a Range of Estimated Baseline Risk Using the Basic Reduced Set

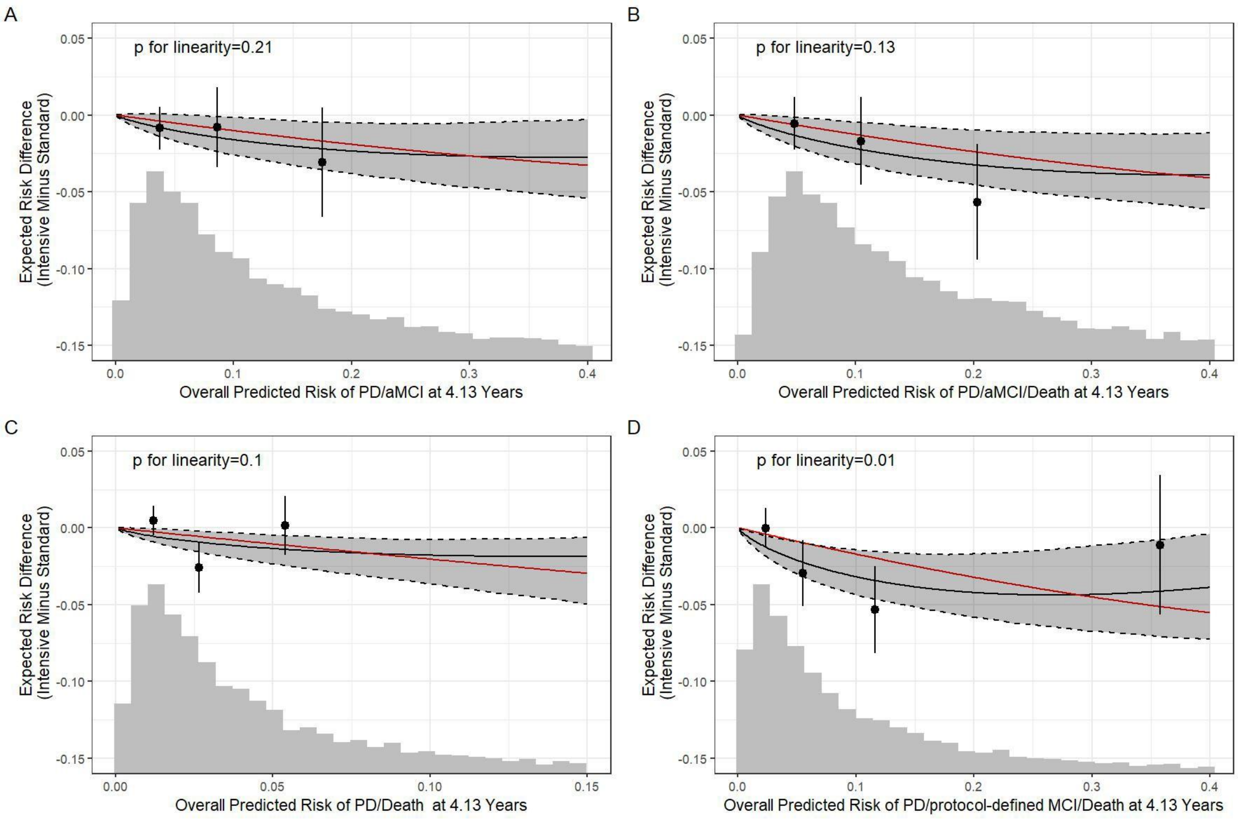

The solid red line represents direct risk magnification, where the absolute risk reduction is directly proportional to the baseline risk assuming a constant relative treatment effect on the hazard ratio scale. The solid black line represents the relationship between average elastic net Cox-model-estimated absolute risk difference for each outcome across the full range of baseline risk. The dashed black line and shaded grey areas represent the 95% confidence bands for the estimated risk difference. The histogram represents the distribution of baseline risk of each outcome in the SPRINT population used in the current analysis. The black circles represent the average observed risk difference for each outcome within quartiles of the Cox-model-estimated absolute risk difference. The bars represent 95% confidence limits of the observed risk differences. Panel A corresponds to risk of probable dementia (PD) or amnestic Mild Cognitive Impairment (aMCI); Panel B corresponds to the risk of PD or aMCI or death; Panel C corresponds to the risk of PD or death; and Panel D corresponds to the risk of PD or protocol defined MCI or death

© 2023 Ghazi L et al. *JAMA Network Open*.
