# Supplementary material for: Identifying Patients for Intensive Blood Pressure Treatment Based on Cognitive Benefit: A Secondary Analysis of the SPRINT Randomized Clinical Trial
Source: JAMA Netw Open. 2023 May 19;6(5):e2314443. doi: 10.1001/jamanetworkopen.2023.14443 (PMC10199351; doi:10.1001/jamanetworkopen.2023.14443)
Supplement: Supplement 3. — Data Sharing Statement [file jamanetwopen-e2314443-s003.pdf]

Data Sharing Statement

Ghazi. Identifying Patients for Intensive Blood Pressure Treatment Based on Cognitive Benefit. *JAMA Netw Open*. Published May 19, 2023. doi:10.1001/jamanetworkopen.2023.14443

Data
Data available: Yes
Data types: Other (please specify)
Additional Information: Data is available at https://biolincc.nhlbi.nih.gov/studies/sprint/
How to access data: https://biolincc.nhlbi.nih.gov/studies/sprint/
When available: With publication

Supporting Documents
Document types: Other (please specify)
Additional Information: Study protocol is attached
How to access documents: Study protocol is attached and will be included with publication
When available: With publication

Additional Information
Who can access the data: Researchers can contact SPRINT PI's for any additional data not available at BioLincc
Types of analyses: The researchers have access to all BioLincc data (no restrictions)
Mechanisms of data availability: The researchers have access to all BioLincc data (no restrictions)
